# Epidemiology of malaria and leishmaniasis in Thailand (2004–2025): A systematic review

**DOI:** 10.1016/j.crpvbd.2026.100386

**Published:** 2026-05-15

**Authors:** Warachaya Apajamjarut, Nalin Siriwongsanon, Patanin Tonpon, Lapatrada Boonlertworakul, Ruamporn Joosiriwong, Baby Kyi Soe, Saowalak Kaewmee, Jassada Saingamsook, Catherine Walton, Sung Jae Lee, Chunqing Lin, George Dimopoulos, Narissara Jariyapan

**Affiliations:** a Faculty of Medicine, Chulalongkorn University, Bangkok, 10330, Thailand; b Center of Excellence in Vector Biology and Vector-Borne Disease, Department of Parasitology, Faculty of Medicine, Chulalongkorn University, Bangkok, 10330, Thailand; c Department of Medical and Public Health Secretary, College of Allied Health Sciences, Suan Sunandha Rajabhat University, Samut Songkhram, 75000, Thailand; d Parasitology and Entomology Research Cluster (PERC), Department of Parasitology, Faculty of Medicine, Chiang Mai University, Chiang Mai, 50200, Thailand; e Department of Earth and Environmental Science, School of Natural Sciences, Faculty of Science and Engineering, University of Manchester, Manchester, M13 9PT, UK; f Department of Psychiatry and Biobehavioral Sciences, Semel Institute for Neuroscience and Human Behavior, University of California, Los Angeles, CA, 90024, USA; g W. Harry Feinstone Department of Molecular Microbiology and Immunology, Bloomberg School of Public Health, Johns Hopkins University, Baltimore, MD, 21205, USA

**Keywords:** Malaria, *Plasmodium*, Leishmaniasis, *Leishmania*, Co-occurrence, Thailand

## Abstract

Malaria and leishmaniasis remain major vector-borne parasitic diseases in tropical and subtropical regions. The aim of this systematic review was to synthesize evidence on the epidemiology and shared ecological and epidemiological characteristics of malaria and leishmaniasis in Thailand from 2004 to 2025. This systematic review was conducted in accordance with PRISMA guidelines and utilized three databases, PubMed, Scopus, and ScienceDirect, supplemented by grey literature and manual reference screening. From 1397 records, a total of 57 studies were included (36 on malaria and 21 on leishmaniasis). Malaria transmission persisted mainly in border and forest-associated areas, with nine *Plasmodium* species documented in Thailand across the included studies, including the emerging zoonotic parasites (*Plasmodium knowlesi* and *Plasmodium cynomolgi*). *Plasmodium vivax* and *Plasmodium falciparum* predominated nationwide; however, *P. falciparum* has shown a declining trend since 2012. Leishmaniasis was reported across multiple regions, with six *Leishmania* species identified. *Leishmania martiniquensis* and *Leishmania orientalis* were predominant species among people living with HIV. Seven provinces had reported occurrences of both diseases across the overall review period, associated with shared ecological conditions, forest-related occupational exposure, and population mobility. However, such findings reflect province-level co-occurrence based on aggregated evidence rather than confirmed concurrent transmission or co-infection. These findings highlight epidemiological characteristics and shared risk factors, particularly among immunocompromised populations and individuals engaged in forest-related activities. Integrated surveillance and control strategies within a One Health framework may strengthen the ability to eliminate malaria transmission and the expanding burden of leishmaniasis in Thailand.

## Highlights

• Malaria transmission persists in border and forest areas in Thailand.
• Thailand shifts toward *P. vivax* predominance and the emergence of zoonotic malaria (*P. knowlesi* and *P. cynomolgi*).
• Asymptomatic reservoirs for both malaria and leishmaniasis are common, with high prevalence noted among PLHIV.
• Seven provinces reported occurrences of both diseases during the review period.
• Shared ecological and occupational risk factors support integrated One Health surveillance.

## Graphical abstract

**Figure:**
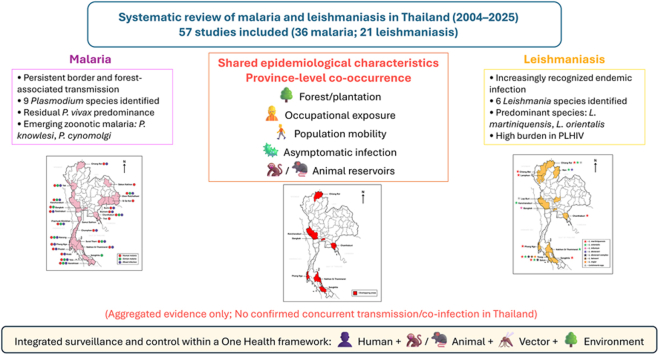

## 1 Introduction

Malaria and leishmaniasis, major parasitic and vector-borne diseases, have raised global health concerns, particularly in tropical and subtropical regions. Malaria is caused by protozoan parasites belonging to the genus *Plasmodium* and transmitted through the infectious bite of female *Anopheles* vector mosquitoes. Traditionally, five *Plasmodium* species, including *P. falciparum*, *P*. *vivax*, *P*. *malariae*, *P. ovalecurtisi* (formerly called *P. ovale*, “classic” form), *P. ovalewallikeri* (formerly called *P. ovale wallikeri*, “variant” form), have been recognized as major causes of human malaria (Snounou et al., 2024). However, increasing evidence has established *P. knowlesi* as an important zoonotic malaria parasite capable of naturally infecting humans, particularly in Southeast Asia (White, 2008). Urban expansion and more convenient and frequent travel have brought humans into closer contact with wildlife by destroying natural habitats, creating shared “edge” environments, and providing easy access to remote areas, which together increase opportunities for pathogens to spillover from wildlife to humans (Hassell et al., 2017; Esposito et al., 2023). Consequently, *P. knowlesi* has emerged as an important zoonotic malaria infection agent affecting humans in recent decades.

The initial symptom of malaria patients is fever. The timing of the fever is irregular depending on malaria parasite species, with some causing fever spikes every 48 h, while others may have a more irregular pattern. Other early symptoms are non-specific, such as chills, headache, myalgia, and gastrointestinal manifestations, making it difficult to distinguish them from other febrile illnesses. Untreated malaria can rapidly progress to severe disease, particularly in infections caused by *P. falciparum*. Severe malaria commonly presents with neurological impairment (e.g. altered consciousness or seizures), profound anemia, respiratory distress, and multi-organ dysfunction, including hepatic and renal failure. In advanced cases, circulatory collapse and extreme weakness may occur (Trampuz et al., 2003).

The spectrum of clinical disease from *P. knowlesi* infection ranges from asymptomatic infection through severe malaria and death. *Plasmodium knowlesi* exhibits a 24-h erythrocytic cycle, resulting in rapid elevations in parasitemia and acute clinical advancement. Although most cases are uncomplicated, severe disease resembling *P. falciparum* malaria may occur. Current treatment in Thailand follows the Clinical Practice Guidelines for the Treatment of Malaria for Physicians, Thailand (Clinical Practice Guidelines, 2025). Malaria treatment depends on the infecting *Plasmodium* species, disease severity, and local epidemiological context. For example, uncomplicated *P. falciparum* malaria is typically treated with artemisinin-based combination therapies (ACTs), while chloroquine followed by primaquine remains effective in uncomplicated *P. vivax* malaria (Clinical Practice Guidelines, 2025). Human malaria has been affecting many countries around the world for centuries. Various strategies have been employed to eliminate malaria, for example, early detection and prompt treatment, mass drug administration campaigns, vector control, surveillance and monitoring, and community engagement. In Thailand, progress has been made but complete elimination of human malaria remains unachieved (Sattabongkot et al., 2022; Oo et al., 2025).

Leishmaniasis, a zoonotic vector-borne disease, is caused by protozoan parasites of the genus *Leishmania*, transmitted through the bites of *Leishmania*-infected sand flies. The disease can manifest in various forms, including cutaneous leishmaniasis (CL), visceral leishmaniasis (VL), and mucocutaneous leishmaniasis (MCL). Currently, over 21 species of *Leishmania* have been documented as human pathogens, and more than 90 sand fly species, mainly phlebotomine sand flies (genus *Phlebotomus* in the Old World and *Lutzomyia* in the New World), are known to transmit *Leishmania* parasites (Hong et al., 2020). Humans are considered incidental hosts, whereas approximately 70 animal species serve as reservoirs for *Leishmania* parasites, for example, dogs, rodents, marsupials, and bats (Azami-Conesa et al., 2021). A combination of strategies, such as integrated vector management (IVM), the introduction of liposomal amphotericin B, and the improvement of welfare, has contributed to the control of leishmaniasis. These strategies can be used by other countries to achieve the same results (Nagi, 2024). However, drug-resistant *Leishmania* parasites have been identified, including miltefosine-resistant *L. donovani* and amphotericin B-resistant *L. amazonensis*. Amphotericin B-resistant *L. martiniquensis* have been documented (Srivastava et al., 2017; Mano et al., 2023a, 2023b; Ferreira et al., 2024; Jariyapan et al., 2025). Additionally, during the past 20 years, four new *Leishmania* spp. have emerged as human pathogens, i.e. *L*. (*Mundinia*) *martiniquensis*, *L*. (*Mundinia*) *orientalis*, *L*. (*Mundinia*) *chancei*, and *L*. (*Leishmania*) *ellisi* (Jariyapan et al., 2018; Kwakye-Nuako et al., 2023; Sapp et al., 2024). Furthermore, no vaccines are available for leishmaniasis, making the disease still a public health problem (WHO, 2023).

Malaria and leishmaniasis may co-occur in settings where their endemic ranges or risk environments overlap, particularly in regions characterized by forest exposure and vector presence. In Yemen and Nepal, malaria and *Leishmania* co-infection cases have been reported (Bin Mohanna, 2015; Ghimire et al., 2019). In Malaysia, reported cases of co-infection have primarily involved imported infections among migrant workers rather than confirmed local transmission of leishmaniasis (Ab Rahman and Abdullah, 2011). It poses an important obstacle to the accurate diagnosis and timely treatment of both diseases. In Thailand, malaria remains a considerable health problem despite the significant progress in malaria control and elimination. Furthermore, indigenous leishmaniasis has been documented in several regions around the country. However, information regarding the shared ecological and epidemiological characteristics of malaria and leishmaniasis in Thailand is limited, despite reports of both diseases in several provinces. Therefore, an updated and comprehensive synthesis of available evidence is needed to better characterize their epidemiology, associated risk factors, and reported occurrence in Thailand.

Therefore, this systematic review aims to (i) synthesize evidence on the prevalence, incidence, and epidemiological characteristics of malaria and leishmaniasis in Thailand between 2004 and 2025; (ii) characterize parasite species diversity, transmission settings, and factors associated with disease occurrence; and (iii) comparatively examine shared ecological and epidemiological characteristics of both diseases, including reported occurrence, at-risk populations, and environmental and occupational risk factors. By integrating findings across multiple study designs, this review seeks to clarify similarities and differences in the epidemiological profiles of malaria and leishmaniasis, identify surveillance gaps, and inform evidence-based strategies for integrated disease monitoring and control in Thailand.

## 2 Materials and methods

### 2.1 Protocol and registration

The review protocol for this systematic review was registered on the Open Science Framework (OSF) (https://doi.org/10.17605/OSF.IO/C36JG). This systematic review was reported in accordance with the Preferred Reporting Items for Systematic Reviews and Meta-Analyses (PRISMA) guidelines (Page et al., 2021).

### 2.2 Search strategy

A comprehensive systematic literature search was undertaken to identify studies reporting the prevalence, epidemiological characteristics, and occurrence of malaria and leishmaniasis in Thailand published between January 2004 and December 2025. The selected period (2004–2025) was chosen to capture the declining trend and epidemiological transition of malaria, and the emergence and increasing recognition of leishmaniasis in Thailand. A structured search strategy was applied across three electronic biomedical databases: PubMed, Scopus, and ScienceDirect. Database searches were conducted between January and February 2026. The search strategy combined free-text keywords and text word searching. (*Leishmania*[tw] OR Malaria[tw] OR *Plasmodium*[tw]) AND (Prevalence[tw] OR Surveillance[tw] OR Case[tw]) AND Thailand[tw] was used to search in PubMed. In Scopus and ScienceDirect, the search strategy was: (*Leishmania* OR Malaria OR *Plasmodium*) AND (Prevalence OR Surveillance OR Case) AND Thailand. To ensure comprehensive coverage, additional sources were consulted, including grey literature, and manual screening of reference lists from relevant articles. This multi-source approach was applied to minimize publication bias and to capture studies not indexed in the selected databases.

### 2.3 Eligibility criteria

Studies were eligible for inclusion if they met the following predefined inclusion criteria: (i) articles published between January 2004 and December 2025; (ii) available in full text; (iii) written in English; (iv) employed observational study designs, including cross-sectional, case-control, and cohort studies (prospective or retrospective), as well as descriptive studies (e.g. case reports), and observational studies encompassing longitudinal and ecological approaches, some of which were based on routine surveillance data, reporting malaria and/or leishmaniasis in Thailand; (v) provided at least one of the following: prevalence of malaria and/or leishmaniasis infection in a defined population, positivity rate among symptomatic patients (with confirmation of active infection), parasite species identified among confirmed cases, diagnostic test, or included information on study location (province or region). Studies were excluded if they were clinical trials, reviews, editorials, or conference abstracts; lacked full-text availability; were published in languages other than English; focused exclusively on insects or animal hosts without human data; or contained overlapping or duplicate datasets.

### 2.4 Study selection

**Fig. 1:**
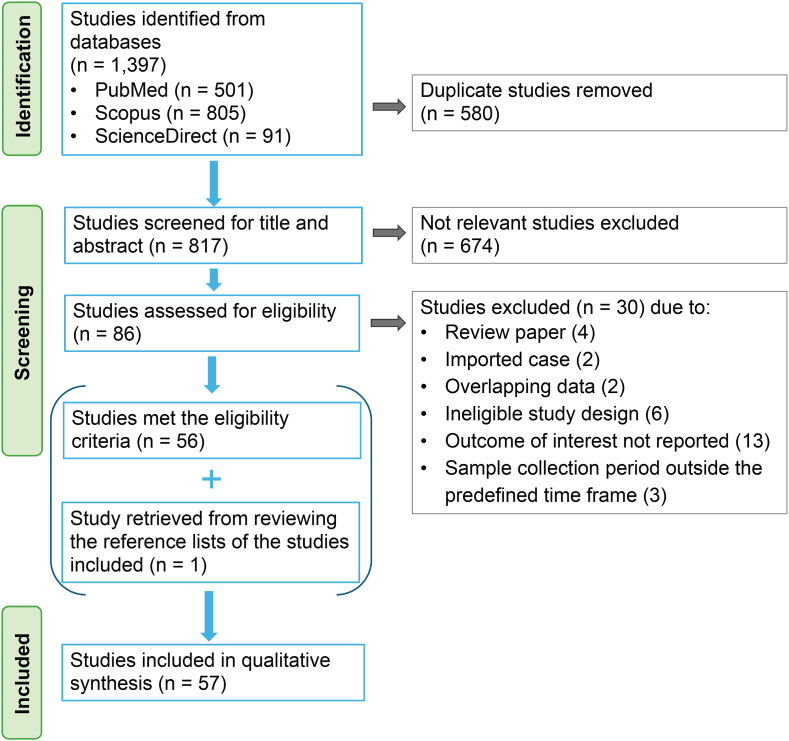
PRISMA flowchart summarizing the literature search strategy and study selection process used in this systematic review.

All identified studies from database searches were imported into Rayyan (https://www.rayyan.ai) (Ouzzani et al., 2016) for reference management; subsequently, duplicates were removed. Five independent reviewers (WA, NS, PT, LB, and RJ) screened titles and abstracts against pre-defined inclusion criteria. Studies deemed eligible proceeded to full-text assessment. Any disagreements were addressed through discussion; when consensus could not be reached, arbitration was provided by senior reviewers (BKS, NJ). The study selection process is summarized in a PRISMA flow diagram (Fig. 1).

### 2.5 Quality assessment

Methodological quality was assessed using the Joanna Briggs Institute (JBI) Critical Appraisal Checklist for case reports (Moola et al., 2020), prevalence studies (Munn et al., 2015), and cohort studies (Moola et al., 2020). Risk of bias assessment was independently conducted by three reviewers (BKS, SK, and NJ). Any discrepancies were resolved through discussion, and study authors were contacted when clarification was required. All appraisal outcomes were recorded using three response options: “Yes,” “No,” or “Unclear.” Each study was evaluated based on key domains of the appraisal tool. An overall score for each study was calculated using the following formula: (Total number of “Yes” responses ÷ Total number of appraisal items) × 100. Based on this score, studies were classified as having a low risk of bias (≥ 70%), a moderate risk of bias (50–69%), or a high risk of bias (< 50%). Studies assessed as having a high risk of bias were excluded from the final synthesis.

### 2.6 Data extraction and qualitative synthesis

To address the review objectives, data were systematically extracted from all included studies using a standardized data extraction form developed in Microsoft Excel. Extracted variables included: (i) type of infection (malaria or leishmaniasis); (ii) period of data collection; (iii) sample size and reported prevalence or incidence; (iv) study location and reported occurrence at regional and provincial levels; (v) characteristics of the study population/case (age, sex, socioeconomic or occupational background, and immune status, where available); and (vi) reported/suggested factors associated with disease occurrence, such as climate conditions, land use, human mobility, and vector control interventions, where available. It should be acknowledged that malaria data represent a robust national reporting system, while leishmaniasis data are heavily weighted toward high-risk groups, i.e. people living with HIV (PLHIV), and specific research sites like Trang Province.

Data extraction was independently performed by five authors (WA, NS, PT, LB, and RJ) and subsequently cross-checked by additional authors (BKS, SK, and NJ) to ensure accuracy and consistency. Results were presented descriptively using summary tables and maps to illustrate reported occurrence patterns and epidemiological trends of malaria and leishmaniasis in Thailand from 2004 to 2025. Where quantitative data pooling was not appropriate because of substantial heterogeneity across studies, results were synthesized qualitatively without statistical aggregation.

## 3 Results

### 3.1 Characteristics of included studies

The initial database search yielded a total of 1397 records. After a full-text review, a total of 57 studies were included in the qualitative synthesis (Supplementary Table S1). Of these, 36 studies investigated malaria and 21 addressed leishmaniasis. While the number of studies for each disease was comparable, the nature of the evidence differed. Malaria research predominantly utilized extensive longitudinal datasets, including longitudinal and ecological studies, and cohort studies, whereas the evidence base for leishmaniasis was more varied, primarily comprising case reports and cross-sectional studies, frequently targeting high-risk populations such as PLHIV.

The included studies comprised a range of observational designs, including case reports (*n* = 24), cross-sectional studies (*n* = 17), case-control studies (*n* = 2), cohort studies (*n* = 9), longitudinal and ecological studies (*n* = 4), and a combined cross-sectional and cohort study (*n* = 1) (Supplementary Table S1). Geographically, the studies covered multiple regions of Thailand, with the largest proportion addressing the northern (*n* = 26) and southern (*n* = 20) regions. Additional studies were conducted in the western (*n* = 7), eastern (*n* = 7), central (*n* = 6), and northeastern (*n* = 5) regions. Overall, the included studies largely focused on areas with sustained malaria transmission and emerging leishmaniasis.

Most included studies were assessed as having low risk of bias, with only one study, Sermwittayawong et al. (2015), classified as moderate risk (Supplementary Tables S2–S4).

### 3.2 Malaria in Thailand (2004–2025)

#### 3.2.1 Study characteristics and parasite diversity

A total of 36 studies were identified from 2004 to 2025, comprising case reports (*n* = 9) of human and zoonotic malaria (Table 1) and 27 studies (Table 2) representing cross-sectional studies (*n* = 13), case-control studies (*n* = 1), cohort studies (*n* = 8), longitudinal and ecological studies (*n* = 4), and a combined cross-sectional and cohort study (*n* = 1) (Supplementary Table S1). Nine species of *Plasmodium*, including *P. falciparum*, *P. vivax*, *P. malariae*, *P. ovalecurtisi* (*P. ovale*, “classic” form), *P. ovalewallikeri* (*P. ovale wallikeri*, “variant” form), *P. inui*, *P. fieldi*, *P. knowlesi*, and *P. cynomolgi*, were reported during the past 20 years (Table 1, Table 2). Five case reports described human malaria infections caused by *P. falciparum* and *P. vivax*, whereas four reports described zoonotic malaria infections, including *P. knowlesi* and *P. cynomolgi* (Table 1).

**Table 1:** Case reports of human and zoonotic malaria infection in Thailand (2004–2025).

ID	Reference [Study year]	Province [Region]	Species	Diagnostic methods	Characteristics of the study case (sex, age, occupation)	Treatment	Reported/suggested factors associated with disease occurrence
**Human malaria**							
1	Lwin et al. (2008) [NR]	Tak [N]	*P. falciparum*	LM	Male; 28 years-old; NR	Artesunate and mefloquine	Residence near Thailand-Myanmar border (forest/border exposure)
2	Luvira et al. (2009) [2008]	Kanchanaburi [W]	*P. falciparum*	LM	Male; 43 years-old; NR	Artesunate and mefloquine	Travel history to a forested area along the Thailand-Myanmar border prior to illness
3	Rijken et al. (2011) [2008]	Tak [N]	*P. vivax*	LM, PCR	Pregnant female; 38 years-old; NR	Chloroquine, dihydroartemisinin-piperaquine and primaquine	Living and working in forested areas along the Thailand-Myanmar border; chloroquine-resistant *P. vivax* malaria
4	Changpradub and Mungthin (2014) [NR]	Sakon Nakhon [NE]	*P. vivax*	LM, PCR	Male; 24 years-old; painter	Artesunate and mefloquine	History of hunting activity in forested areas (Pupan Mountain) in Sakon Nakhon Province
5	Boonyarangka et al. (2022) [2021]	Ratchaburi [C]	*P. vivax*	RDT, PCR	Male; 25 years-old; resort worker	Chloroquine and primaquine	Forest exposure related to mushroom collection in Ratchaburi Province; COVID-19 co-infection
**Zoonotic malaria**							
6	Jongwutiwes et al. (2004) [2000]	Bangkok [C]	*P. knowlesi*	LM, PCR	Male; 38 years-old; NR	Chloroquine	History of stay in a hilly forest area in Prachuap Khiri Khan Province near the Thailand-Myanmar border; first reported human *P. knowlesi* case in Thailand
7	Nakaviroj et al. (2015) [2013]	Chanthaburi [E]	*P. knowlesi*	LM, PCR	Male; 58 years-old; NR	Artesunate	Camping in forested national park, Khao Khitchakut National Park in Chantaburi Province, bordering Cambodia; reported mosquito exposure and presence of wild long-tailed macaques
8	Ngernna et al. (2019) [2017–2018]	Songkhla [S]	*P. knowlesi*	LM, PCR	Male; 22 years-old; rubber plantation worker	Chloroquine and primaquine	Camping out in a forest area in Ban Na Kha, Malaysia, surrounded by a wooded area populated by wild macaques
		Narathiwat [S]	*P. knowlesi*	RDT, PCR	Male; 45 years-old; palm plantation worker	Chloroquine and primaquine	Travel to forested areas in Malaysia inhabited by macaques
		Songkhla [S]	*P. knowlesi*	LM, PCR	Male; 50 years-old; rubber plantation worker	Chloroquine and primaquine	Cross-border forest travel (Thailand-Malaysia border)
		Songkhla [S]	*P. knowlesi*	Unspecified, PCR	Female; 48 years-old; rubber plantation worker and non-timber forest product finder	Chloroquine and primaquine	Cross-border forest travel (Thailand-Malaysia border)
		Songkhla [S]	*P. knowlesi*	LM, PCR	Male; 32 years-old; rubber plantation worker and non-timber forest product finder	Dihydroartemisinin-piperaquine and primaquine	Cross-border forest exposure in Malaysia (macaque-inhabited areas)
		Songkhla [S]	*P. knowlesi*	LM, PCR	Male; 35 years-old; rubber plantation worker and wild animal hunter	Chloroquine and primaquine	Cross-border forest travel (Thailand-Malaysia border)
9	Sai-ngam et al. (2022) [2021]	Yala [S]	*P. cynomolgi*	LM, Singleplex RT-PCR	Female; 53 years-old; rubber plantation worker	Chloroquine and primaquine	NR
			*P. cynomolgi*	LM, Singleplex RT-PCR	Female; 55 years-old; rubber plantation worker	Chloroquine and primaquine	NR
			*P. cynomolgi* co-infection with *P. vivax*	LM, Singleplex RT-PCR, Multiplex RT-PCR	Male; 25 years-old; Royal Thai Army	Chloroquine and primaquine	Prolonged forest patrol and overnight stays (military personnel); forest exposure

*Abbreviations*: ID, identification number; LM, light microscopy; NR, not reported; PCR, polymerase chain reaction; RDT, rapid diagnostic test; RT-PCR, real-time polymerase chain reaction; [C], Central region; [E], Eastern region; [N], Northern region; [S], Southern region; [W], Western region.

**Table 2:** Prevalence, incidence, epidemiological characteristics, and factors associated with the occurrence of malaria in Thailand (2004–2025).

ID	Reference [Study year]	Province [Region]	Population examined/Confirmed cases (*n*) or incidence data	Species distribution (% Prevalence or incidence)	Diagnostic methods	Characteristics of the study population (sex, age, occupation, immune status)	Reported/suggested factors associated with disease occurrence
**Cross-sectional study**							
11	Putaporntip et al. (2009) [2006–2007]	Tak [N]; Chanthaburi [E]; Prachuab Khirikhan [S]; Yala [S]; Narathiwat [S]	1874 febrile patients examined; 1751 malaria-positive cases (93.4%)	*P. falciparum* (30.7%), *P. vivax* (54.0%), *P. malariae* (0.20%), *P. ovale* (0.17%), *P. knowlesi* (0.60%, including mixed infections)	LM, PCR	Predominantly male (2.25:1); age 1–81 years (mean 25.5); Thai (56%) and cross-border migrants from Myanmar (36%) and Cambodia (8%)	Cross-border migration; seasonal variation; residence in macaque-inhabited areas (*P. knowlesi* cases)
13	Kritsiriwuthinan and Ngrenngarmlert (2011) [2008]	Bangkok [C]; Samut Sakhon [C]	294 participants	*Plasmodium* spp. (1.36%)	LM	Males and females; age 9–46 years (median 26); predominantly Myanmar migrants	All positive cases were male Myanmar workers; no significant association with age, gender, or province
14	Kitvatanachai and Rhongbutsri (2012) [2008]	Kanchanaburi [W]	671 asymptomatic migrant workers and 38 symptomatic patients	Asymptomatic: 1.64%, predominantly *P. vivax* (81.8%); Symptomatic: 35.7%, predominantly *P. falciparum* (80.0%)	LM	Asymptomatic: age 16–42 years, Female:Male (1:1.3); symptomatic: age 13–36 years, Female:Male (1.4:1)	The majority of infections linked to migrants from the Mohlumyai area, Myanmar
15	Sermwittayawong et al. (2012) [2008–2010]	Ranong [S]	419 participants (171 Thai and 248 Myanmar)	*P. falciparum* (45.35%), *P. vivax* (53.22%), mixed infection (*P. falciparum* + *P. vivax*) (0.95%), *P. knowlesi* (0.48%) by PCR	LM, PCR, DNA sequencing	Two *P. knowlesi* cases (45-year-old Thai male and 30-year-old Myanmese male), both worked in Kawthoung district, Myanmar	Cross-border transmission in the Thai-Myanmar border area
16	Baum et al. (2015) [2012]	Tak [N]	219 community samples; 61 clinical patients	Community (qPCR): *Plasmodium* sp. (11.4%), *P. vivax* (7.7%), *P. falciparum* (3.65%); Clinic (qPCR): *Plasmodium* sp. (44.3%), *P. vivax* (21.3%), *P. falciparum* (11.5%), mixed *P. vivax* + *P. falciparum* (11.5%)	LM, qPCR	Community residents (asymptomatic survey) and symptomatic clinic patients	NR
19	Sermwittayawong et al. (2015) [2009–2010]	Ranong [S], Yala [S]	408 microscopy-confirmed malaria cases	Ranong: *P. falciparum* (46.00%), *P. vivax* (54.00%); Yala: *P. falciparum* (52.00%), *P. vivax* (45.00%)	LM, Single nucleotide polymorphism (SNP) analysis of codon 86 in the pfmdr1 gene	NR	Marked geographical difference in pfmdr1 N86Y mutation frequency between Ranong and Yala
20	Sriwichai et al. (2017) [2011–2014]	Tak (Thailand-Myanmar border) [N]	621 microscopy-confirmed malaria cases	*P. falciparum* (43.00%), *P. vivax* (57.00%)	LM	Mixed Thai residents and migrants (M1, M2); median age 13 years; 47% male	*P. falciparum* is associated with recent migration (imported); *P. vivax* is associated with local vector density
21	Sattabongkot et al. (2018) [2015]	Tak [N]	3650 community residents examined; 85 sub-microscopic *Plasmodium* infections confirmed by LAMP and PCR (2.33%)	*P. vivax* (52.9%), *P. falciparum* (30.6%), *P. malariae* (2.4%), mixed *P. falciparum* + *P. vivax* (4.7%), *Plasmodium* spp. undetermined (9.4%)	LM, LAMP	Male and female; age 1 month to 99 years (median 15); predominantly asymptomatic; higher prevalence in age 31–45 years; plantation workers and merchants at increased risk	Asymptomatic sub-microscopic carriage; plantation and merchant occupations; specific hamlets (Nong Bua, Suan Oi); molecular detection revealed substantially higher prevalence than microscopy
28	Chaivisit et al. (2020) [2013–2016]	Chumphon [S], Nakhon Si Thammarat [S], Krabi [S], Phang Nga [S], Phuket [S], Ranong [S], Surat Thani [S]	4244 new malaria cases	*P. falciparum* (60.13%)*, P. vivax* (38.15%)*, P. malariae* (0.94%)*,* mixed *P. falciparum* + *P. vivax* (0.78%)	Routine national malaria surveillance database	Thai nationals; highest incidence in males aged 15–44 years; 61.73% rubber agricultural workers	Incidence associated with district, sex-age category, and year; seasonal peak (May-June); high-risk border districts near Myanmar; land-use change/deforestation
29	Shimizu et al. (2020) [Jan-May 2019]	Surat Thani [S]	9418 participants; 7034 samples (Jan) and 8671 samples (May); Infections: 0.45% in January and 0.61% in May	Combined infections: 85/15,705 (0.54%); *P. falciparum* (34%), *P. vivax* (34%), *P. knowlesi* (3.5%), unknown (28%)	RDT, PCR, qPCR	Thai nationals; median age 39 years; 82.6% ≥ 15 years; 50.9% female; majority agriculture-related; mostly asymptomatic infections	Staying outdoors at night-time; male sex; seasonal peak (May); predominantly outdoor transmission
30	Putaporntip et al. (2021) [2007–2018]	Tak [N], Ubon Ratchathani [NE], Chanthaburi [E], Yala [S], Narathiwat [S]	1359 febrile patients tested; 1180 PCR-confirmed malaria cases; *P. cynomolgi* co-infections (0.76% of PCR-positive cases)	*P. vivax* (69.66%)*, P. falciparum* (23.14%)*, P. knowlesi* (0.34%), *P. malariae* (0.25%), *P. ovale* (0.09%), mixed *P. vivax* + *P. falciparum* (4.66%), mixed *P. vivax* + *P. knowlesi* (0.76%), mixed *P. vivax* + *P. cynomolgi* (0.59%), mixed *P. vivax* + *P. knowlesi* + *P. cynomolgi* (0.09%), mixed *P. falciparum* + *P. knowlesi* (0.34%), mixed *P. falciparum* + *P. cynomolgi* (0.09%)	LM, PCR, DNA sequencing	Febrile patients attending malaria clinics/hospitals, age 7–85 years, 79.7% male, all symptomatic; most *P. cynomolgi* cases were male (8/9), age 15–53 years, some lived near domesticated/wild macaques	Suggested zoonotic transmission from macaques (long-tailed and pig-tailed); residence in proximity to macaques; more cases during the rainy season; morphological similarity with *P. vivax* may cause misdiagnosis; cross-species transmission likely *via* shared *Anopheles* vectors
31	Putaporntip et al. (2022) [1996–2018]	Tak [N], Ubon Ratchathani [NE], Chanthaburi [E], Yala [S], Narathiwat [S]	5271 acute febrile patients tested; 4195 PCR-confirmed malaria cases; simian malaria detected in 68 patients (1.62% of PCR-positive cases)	Human malaria species present: *P. vivax* (60.50%), *P. falciparum* (28.03%), *P. malariae* (0.1%), *P. ovale curtisi* (0.02%), *P. ovale wallikeri* (0.02%); Simian species: *P. knowlesi* (0.72%), *P. cynomolgi* (0.50%), *P. inui* (0.45%), *P. fieldi* (0.07%); Mixed infections: 10.92% double; 0.17% triple; 85.29% of simian infections were mixed (mostly with *P. vivax*)	LM, PCR, DNA sequencing	Symptomatic febrile patients attending malaria clinics/hospitals; age 1–90 years (mean 31.2 years); 69.65% male; most simian malaria cases were co-infections; *P. inui* patients aged 4–54 years (mean 29.6; Male:female ratio 2.17:1); *P. fieldi* detected only in Yala (2017–2018); many patients reported residence near macaques	Suggested zoonotic transmission from long-tailed and pig-tailed macaques; higher occurrence during rainy season; residence near macaque habitats; likely vector-mediated cross-species transmission; microscopy frequently misdiagnosed as simian species as *P. vivax* or *P. falciparum*
36	Aung et al. (2025b) [Mar-Apr 2025]	Tak [N]	300 short-term Myanmar migrants; 6 PCR-confirmed cases (2% prevalence)	*P. vivax* (100%)	Genus-specific qPCR, species-specific qPCR	Age 18–78 years; 52.7% female; 71.3% Karen ethnicity; 71% no formal education; 39% daily wage labourers; all informal employment; 39% reported prior malaria; all infections asymptomatic (afebrile)	Lower malaria knowledge; lower care-seeking score; majority of infected individuals were Karen ethnicity; daily wage labourers; low education level; frequent return to Myanmar (descriptive pattern); asymptomatic carriage
**Cross-sectional study and cohort study**							
12	Jongwutiwes et al. (2011) [2008–2009]	Tak [N]; Chantaburi [E]; Yala [S]; Narathiwat [S]	3770 febrile patients examined; 3446 PCR-positive (91.4%)	*P. falciparum* (45.15%), *P. vivax* (41.27%), *P. malariae* (0.15%), *P. ovale* (rare), *P. knowlesi* detected in 23 cases (0.67%, 8 monoinfections and 15 co-infections), mixed-species infections 13.26%	LM, PCR, DNA sequencing	Predominantly male (∼2:1)	Regional and seasonal variation; higher mixed-species infections in specific provinces; *P. knowlesi* associated with residence in macaque-inhabited areas
**Case-control study**							
10	Chaveepojnkamjorn and Pichainarong (2004) [2002]	Chiang Rai [N]; Kanchanaburi [W]	217 confirmed malaria cases	*P. falciparum* (60.8%), *P. vivax* (36.4%), mixed infection (*P. falciparum* + *P. vivax*) (2.8%)	LM	Predominantly male; majority < 35 years; Thai-Yai and Myanmar ethnic groups	Forest residence and workplace; outdoor exposure; population mobility; low personal protection
**Cohort study**							
22	Lawpoolsri et al. (2019) [2011–mid-2017]	Tak [N]	7812/410 (5.2% cumulative incidence over 6.5 years; 527 malaria episodes)	*P. vivax* (64.5%, 340/527 episodes), *P. falciparum* (34.3%, 181/527 episodes), Mixed infection (1.1%, 6/527 episodes)	LM, RDT	Male and female; all age groups (< 5 years to > 60 years); open community-based cohort; symptomatic and asymptomatic infections; higher recurrence among males and younger age groups (especially 6–10 years); Karen ethnicity predominant; various occupations	Male sex; young age (especially 6–10 years); Karen ethnicity; forest-related occupation; child/student status; factory and plantation work; presence of other malaria-infected household members during the same period; clustering within households; shorter recurrence interval in younger individuals; majority of recurrent episodes caused by the same *Plasmodium* species
25	Nguitragool et al. (2019) [2013–2014]	Kanchanaburi [W], Ratchaburi [C]	999 participants (prospective cohort; 735 PCR-detected infections)	*P. vivax* (1.7–4.2%), *P. falciparum* (0–1.3%); mixed infection (6.3% of genotyped infections)	qPCR	Male and female; age 1–83 years (mean 23 years); predominantly agricultural border population (Thai and Karen); majority asymptomatic	Malaria risk associated with rainy season, agriculture, male sex, previous clinical malaria, and travel to Myanmar; indoor residual spray (IRS) protective; infections are highly clustered with substantial asymptomatic reservoir
27	Yorsaeng et al. (2019) [2013–2014]	Kanchanaburi [W]	812 participants	*P. malariae* (0.25%)	qRT-PCR	Rural village population (∼800 residents); Thai and Karen ethnic groups; agriculture main occupation; 1 asymptomatic chronic infection (41-year-old male); 1 clinical case (54-year-old female)	Low-density persistent asymptomatic infection; misidentification by microscopy; potential chronic reservoir sustaining transmission at very low prevalence; challenge for malaria elimination
33	Gilder et al. (2025) [2012–2015]	Tak [N]	4352 asymptomatic pregnant women	*P. vivax* (59.8%), *P. falciparum* (6.5%), mixed *P. falciparum* + *P. vivax* (1.8%), unspeciated (31.9%)	LM, qPCR, species-specific nested PCR	Migrant and refugee pregnant women attending first ANC visit; median gestational age 16.5 weeks; largely asymptomatic; low seasonal transmission setting; regular biweekly malaria screening	sMiP associated with a high risk of subsequent microscopic malaria and reduced birth weight; *P. falciparum* sMiP associated with maternal anaemia
**Cohort (retrospective) study**							
18	Rattanapunya et al. (2015) [2005–2013]	Tak [N]	867 confirmed malaria cases	*P. falciparum* (54.80%), *P. vivax* (40.40%), *P. malariae* (0.20%), mixed *P. falciparum* + *P. vivax* (4.60%)	LM	Predominantly Burmese migrants; most cases aged 19–40 years; all symptomatic	HIV-malaria co-infection (prevalence 1.85%)
23	Kotepui et al. (2019) [2012–2018]	Tak [N]	971 confirmed malaria cases/95 recurrent cases (9.78%)	*P. vivax* (74.5%), *P. falciparum* (25.5%)	LM	Male and female; age 1–91 years (mean 17.5–20 years); Thai (58.1%) and non-Thai (41.9%); majority students and farmers; cases from OPD and IPD; higher recurrence among males and the student group	Male sex; Thai nationality; student occupation; presence of headache; hematological changes after initial drug regimen (decreased neutrophils, decreased RBC/Hb, increased lymphocytes, increased platelets); recurrence associated with specific demographic and clinical characteristics
26	Saita et al. (2019) [2012–2017]	Tak [N], Kanchanaburi [W]	Provincial population (2012–2017); *P. falciparum* cases: 6454 (2012) to 106 (2017); *P. vivax* cases: 9480 (2012) to 1250 (2017) (routine surveillance)	*P. falciparum* and *P. vivax* (API marked decline 2012–2017; 65.22% and 40.99% of sub-districts at elimination level for *P. falciparum* and *P. vivax*, respectively)	Routine surveillance data (microscopy in hospitals/clinics; RDT in malaria posts)	Predominantly Thai; majority cases aged 5–14 years (Tak) and 25–44 years (Kanchanaburi); common among agricultural workers; migrants (M1/M2)	Spatial clustering along border areas; seasonal peaks (rainy season); cross-border movement and migration; agricultural/forest-related exposure; persistent transmission foci acting as reservoirs; risk of re-importation
**Longitudinal and ecological study**							
17	Kaewpitoon et al. (2015) [2008–2012]	Buriram [NE], Surin [NE]	846 reported malaria cases	*P. vivax* (45.36%), *P. falciparum* (40.66%), mixed infection (8.66%), unidentified (5.32%)	Secondary data from routine surveillance system (diagnostic method not specified)	Predominantly males; most cases aged 31–40 years; mainly agricultural workers	Malaria morbidity is associated with agricultural land use, population density, *Anopheles* adult density, and average annual relative humidity
24	Mercado et al. (2019) [2012–2015]	Tak [N]	526,045–618,382 (annual population); 36,536 confirmed cases (2012–2015, routine surveillance)	*P. vivax* (61.08%), *P. falciparum* (35.98%), *P. malariae* (0.12%), mixed *P. falciparum* + *P. vivax* (0.42%), unknown (2.40%)	Routine national malaria surveillance (microscopy-based confirmation; BVBD database)	Male predominance; majority ≥ 15 years; Myanmar and Thai nationals; cases concentrated along Thai-Myanmar border districts	Rainfall associated with total and *P. vivax* annual parasite incidence per 1000 population (API), higher in forested sub-districts
32	Pongsoipetch et al. (2024) [2011–2021]	Si Sa Ket [NE], Ubon Ratchathani [NE]	23,570 confirmed malaria cases included (Si Sa Ket *n* = 7825; Ubon *n* = 15,745)	API for *P. vivax* was higher than *P. falciparum*; Total confirmed case decline from 1061 in 2011 (*P. vivax* = 881, *P. falciparum* = 180) to 36 in 2021 (*P. falciparum* = 4 confirmed cases in last 2 years)	Secondary data from confirmed malaria cases by LM, RDT	Individual anonymized records of malaria cases reported between 2011 and 2021 in Si Sa Ket and Ubon Ratchathani obtained from the DVBD and actively detected cases from the investigation of transmission foci, which has been performed in Thailand since 2009	Forest proximity and forest cover; cross-border mobility; mobile/migrant populations; agricultural work, rubber plantations, cassava plantations, and forest-going activities; 2014–2016 forest-related outbreak (rosewood harvesting); environmental/climatic factors
34	Pratumchart et al. (2025) [2012–2018]	77 provinces (nationwide)	31,668 confirmed malaria cases; Incidence decreased from 2.16 to 0.84 per 10,000 (*P. vivax*) and 2.16 to 0.13 per 10,000 (*P. falciparum*) between 2012 and 2018	*P. vivax* (∼87.6%), *P. falciparum* (∼12.2%), mixed *P. falciparum* + *P. vivax* (∼0.1%)	Routine national malaria surveillance (microscopy-based and/or RDT confirmation; BVBD database)	Not individually described (secondary surveillance dataset; population-level district data, nationwide coverage)	Seasonal pattern observed; environmental and climatic factors significantly associated: high-risk clusters along Thailand-Cambodia and Thailand-Myanmar borders; central Thailand low risk
35	Aung et al. (2025a) [2018–2023]	Tak [N]	13,347 febrile malaria-suspected patients; 1541 confirmed cases	*P. vivax* (95.5.%), *P. falciparum* (3.2%), *P. malariae* (1.0%), mixed *P. falciparum* + *P. vivax* (0.2%)	LM (WHO protocol); two expert confirmations	Febrile individuals presenting to malaria clinics; majority Thai (89.2%); 57.6% male; high proportion of school-aged children (32.2%); border population with frequent cross-border movement	Age 5–14 years and 15–34 years; male; Myanmar nationals, other non-Thai nationals; recent travel cross-border within 2 weeks; livestock occupation

*Abbreviations*: ID, Identification number; LM, Light microscopy; LAMP, Loop-mediated isothermal amplification; NR, Not reported; PCR, Polymerase chain reaction; qPCR, quantitative PCR; qRT-PCR, quantitative reverse transcription PCR; RDT, rapid diagnostic test; [C], Central region; [E], Eastern region; [N], Northern region; [NE], Northeastern region; [S], Southern region; [W], Western region; DVBD, the Division of Vector-borne Diseases, Department of Disease Control, Ministry of Public Health, Thailand; sMiP, submicroscopic malaria in pregnancy.

#### 3.2.2 Prevalence and incidence patterns

Longitudinal and ecological studies (*n* = 4), and cohort studies (*n* = 8) consistently demonstrated a marked reduction in malaria incidence over time. Earlier studies (2004–2012) (*n* = 9) frequently reported *P. falciparum* as the dominant or co-dominant species. National data from 2012 to 2018 showed a marked decline in *P. falciparum* incidence, while *P. vivax* became increasingly predominant (Pratumchart et al., 2025). Provincial analyses in Tak, Kanchanaburi, Si Sa Ket, and Ubon Ratchathani similarly reported sharp reductions in confirmed cases between 2011 and 2021, accompanied by a substantial reduction in *P. falciparum* cases. Nevertheless, *P. vivax* persisted as the dominant residual species in elimination-phase settings (Pongsoipetch et al., 2024). Cross-sectional and cohort studies revealed heterogeneous prevalence depending on population type and diagnostic method. Community-based surveys generally reported low prevalence (< 1–3%) by microscopy, whereas molecular methods (PCR, qPCR, LAMP) detected higher rates, including submicroscopic infections ranging from approximately 2% to > 10% in some border communities (Baum et al., 2015; Sattabongkot et al., 2018; Yorsaeng et al., 2019). In contrast, clinic-based febrile populations showed substantially higher positivity rates, often exceeding 40–90% among suspected malaria cases in earlier border-focused studies. Cohort studies further documented cumulative incidence ranging from approximately 5% over multi-year follow-up, with frequent recurrent episodes, particularly due to *P. vivax* (Table 2).

Mixed-species infections were regularly reported, although typically at low proportions (0.1–13.0%). Zoonotic malaria was increasingly reported during the study period. The first documented human *P. knowlesi* infection in Thailand was reported in 2004 (Jongwutiwes et al., 2011). Subsequent molecular-based studies identified additional cases of *P. knowlesi*, as well as *P. cynomolgi*, *P. inui*, and *P. fieldi*, which often occurred as mixed infections with *P. vivax*. Simian malaria accounted for approximately <1.0–1.6% of PCR-confirmed cases in multi-province analyses and was frequently misdiagnosed as *P. vivax* or *P. falciparum* by microscopy (Putaporntip et al., 2021, 2022).

#### 3.2.3 Geographical occurrence and epidemiological patterns

**Fig. 2:**
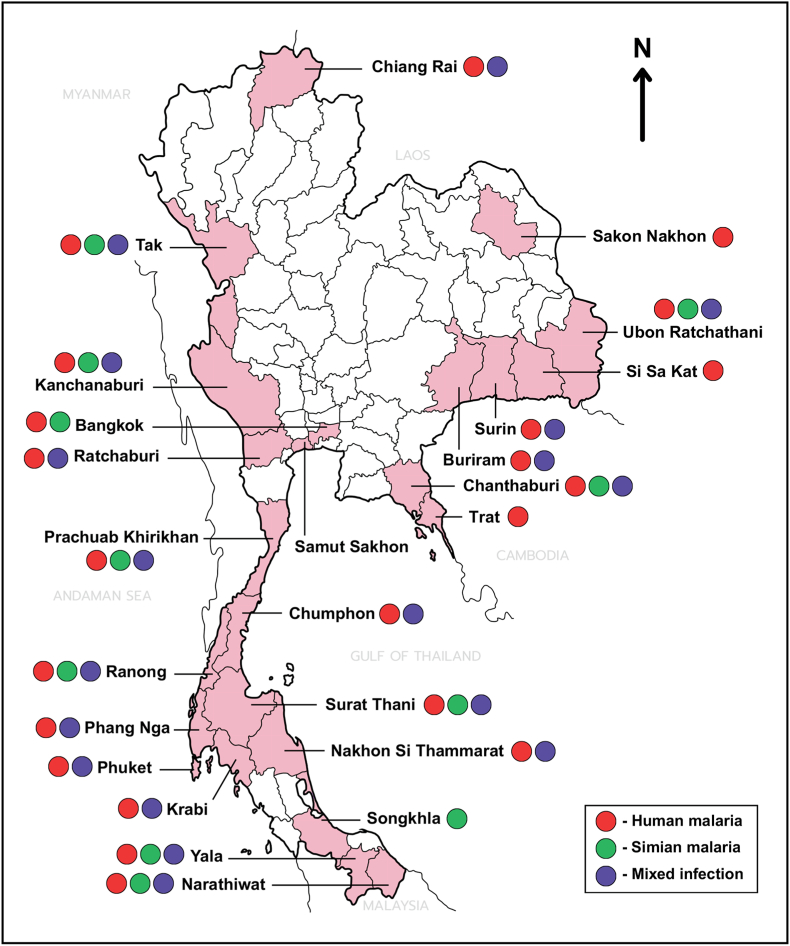
Occurrence of *Plasmodium* species reported in Thailand from 2004 to 2025 according to data presented in Table 1, Table 2 The map summarizes province-level findings from included malaria studies and illustrates reported occurrence of human malaria species, simian malaria parasites, and mixed infections.

Although surveillance systems operate nationwide, malaria occurrence during 2004–2025 was geographically heterogeneous and predominantly localized in areas adjacent to international borders. Province-level data consistently demonstrated spatial clustering along international borders, particularly the Thailand-Myanmar and Thailand-Cambodia borders. High-burden provinces repeatedly identified across multiple study designs included Tak, Kanchanaburi, Chanthaburi, Yala, Narathiwat, Ranong, and several southern provinces (e.g. Surat Thani, Nakhon Si Thammarat, Phang Nga). Zoonotic malaria, specifically *P. knowlesi* and *P. cynomolgi*, was progressively documented in the southern provinces next to Malaysia, including Yala, Narathiwat, and Songkhla (Jongwutiwes et al., 2011; Putaporntip et al., 2021, 2022). The northeastern provinces exhibited significant reductions in incidence over the past decade, with some districts achieving elimination-level transmission (Pongsoipetch et al., 2024). The province-level occurrence of human malaria species, simian malaria parasites, and mixed infections reported in Thailand from 2004 to 2025 is shown in Fig. 2.

#### 3.2.4 Factors associated with malaria occurrence

Malaria occurrence in Thailand was associated with multiple demographic, behavioral, environmental, and ecological factors across the included studies. Host-related factors reported in epidemiological analyses included male sex, younger age groups, student status, agricultural and plantation occupations, migrant status, and a history of previous malaria episodes. Recurrent malaria was more common among males, students, and individuals infected with *P. falciparum* in certain cohorts (Lawpoolsri et al., 2019). HIV-malaria co-infection was reported in border provinces and was associated with higher parasite density and anemia (Rattanapunya et al., 2015). Behavioral and occupational exposures consistently identified across studies included forest-related work, rubber plantation employment, hunting, mushroom collection in forested areas, military patrol activities, and overnight stays in forests (Changpradub and Mungthin, 2014; Boonyarangka et al., 2022; Sai-ngam et al., 2022). Outdoor exposure was significantly associated with malaria infection in several studies (Chaveepojnkamjorn and Pichainarong, 2004; Shimizu et al., 2020). Population mobility and migration were frequently reported, particularly along the Thailand-Myanmar, Thailand-Cambodia, and Thailand-Malaysia borders (Chaveepojnkamjorn and Pichainarong, 2004; Putaporntip et al., 2009; Sriwichai et al., 2017; Saita et al., 2019; Pongsoipetch et al., 2024). Reported environmental and ecological factors associated with malaria included proximity to forests, degree of forest cover, agricultural land use, relative humidity, and rainfall (Mercado et al., 2019; Nguitragool et al., 2019; Pongsoipetch et al., 2024). Studies of zoonotic malaria reported associations with residence or occupational activities in areas inhabited by long-tailed and pig-tailed macaques. Molecular investigations confirmed the presence of simian malaria parasites in humans in these areas (Putaporntip et al., 2009, 2021, 2022; Jongwutiwes et al., 2011; Nakaviroj et al., 2015; Ngernna et al., 2019).

### 3.3 Leishmaniasis in Thailand (2004–2025)

#### 3.3.1 Study characteristics and parasite diversity

In this review, the documented cases of autochthonous leishmaniasis in Thailand between 2004 and 2025, consisting of case reports (*n* = 15) (Table 3), cross-sectional studies (*n* = 4), case-control studies (*n* = 1), and cohort studies (*n* = 1) (Table 4), were identified. A total of six different species of *Leishmania* were reported, which included *L*. *martiniquensis*, *L*. *orientalis*, *L*. *infantum*, *L*. *donovani*, *L. lainsoni*, and *L*. *major*, as well as undetermined *Leishmani*a species.

**Table 3:** Case reports of autochthonous leishmaniasis in Thailand (2004–2025).

ID	Reference [Study year]	Province [Region]	Species [Clinical form]	Diagnostic methods	Characteristics of the study case (sex, age, occupation, immune status)	Treatment/Relapse	Reported/suggested factors associated with disease occurrence
37	Kongkaew et al. (2007) [2005]	Nan [N]	*Leishmania* spp. [VL]	LM	Male; 40 years-old; Construction worker; Immunocompetent	Amp B/No	Sand files, *Sergentomyia* spp.; DAT positive in 1 cat and 3 cows
38	Maharom et al. (2008) [2007]	Bangkok [C]	*L*. *infantum* [VL]	LM, DAT, PCR, DNA sequencing	Male; 66 years-old; Lumber truck driver; Immunocompetent	Amp B/No	NR
39	Sukmee et al. (2008) [2006]	Phang Nga [S]	*L*. *martiniquensis* (identified as *L*. *siamensis*) [VL]	LM, DAT, PCR, DNA sequencing	Male; 55 years-old; Rubber planter; Immunocompetent	Amp B/Yes	NR
40	Suankratay et al. (2010) [2009]	Chanthaburi [E]	*L*. *martiniquensis* [VL]	LM, PCR, DNA sequencing	Male; 37 years-old; Fisherman: HIV with CD4^+^ = 129 cells/mm^3^	Amp B and itraconazole/No	NR
41	Bualert et al. (2012) [2010]	Trang [S]	*L*. *orientalis* (identified as *L*. *siamensis*) [CL, VL]	LM, PCR, DNA sequencing	Female; 32 years-old; NA; HIV with CD4^+^ = 107 cells/mm^3^	Amp B and fluconazole/NA	NR
42	Chusri et al. (2012) [2011]	Songkhla [S]	*L*. *martiniquensis* [CL, VL]	LM, PCR, DNA sequencing	Male; 46 years-old; Rubber planter; HIV with CD4^+^ = 175 (cells/mm^3^)	Amp B and itraconazole/Yes	NR
		Trang [S]	*L*. *martiniquensis* [CL, VL]	LM, PCR, DNA sequencing	Male; 30 years-old; Pet store owner; HIVwith CD4^+^ = 111 cells/mm^3^	Amp B and itraconazole/Yes	NR
43	Kattipathanapong et al. (2012) [2011]	Lop Buri [C]	*Leishmania* spp. [CL]	LM	Female; 3 years-old; NA; Immunocompetent	Itraconazole/No	NR
44	Phumee et al. (2013) [2012]	Chiang Rai [N]	*L*. *martiniquensis* [CL]	LM, PCR, DNA sequencing	Male; 45 years-old; NA; HIV with CD4^+^ < 50 cells/mm^3^	NA/NA	NR
45	Osatakul et al. (2014) [2010]	Satun [S]	*L*. *martiniquensis* [VL]	LM, DAT, PCR, DNA sequencing	Female; 7 years-old; NA; Immunocompetent	Amp B/Yes	NR
46	Pothirat et al. (2014) [2012]	Lamphun [N]	*L*. *martiniquensis* [VL]	LM, PCR, DNA sequencing	Male; 52 years-old; Farmer; Immunocompetent	Amp B/Yes^a^	NR
47	Chiewchanvit et al. (2015) [2013]	Chiang Mai [N]	*L*. *martiniquensis* [CL, VL]	LM, PCR, DNA sequencing	Male; 48 years-old; Salesperson; HIV, CD4^+^ = 121 cells/mm^3^	Amp B and itraconazole/Yes	NR
		Lamphun [N]	*L*. *martiniquensis* [CL, VL]	LM, PCR, DNA sequencing	Male; 38 years-old; Lumberjack; HIV, CD4^+^ = 543 cells/mm^3^	Amp B and itraconazole/No	NR
48	Supsrisunjai et al. (2017) [2015]	Kanchanaburi [W]	*L*. *orientalis* [CL]	LM, PCR, DNA sequencing	Female; 42 years-old; Housewife; HIV, CD4^+^ = 89 cells/mm^3^	Amp B and itraconazole/No	NR
49	Jariyapan et al. (2018) [2014]	Nan [N]	*L*. *orientalis* [CL]	LM, PCR, DNA sequencing	Female; 57 years-old; Gardener; Immunocompetent	Amp B and fluconazole/No	NR
50	Srivarasat et al. (2022) [2021]	Chiang Rai [N]	*L*. *martiniquensis* [CL, MCL, VL]	LM, PCR, DNA sequencing	Female; 47 years-old; NA; HIV, CD4^+^ = 185 cells/mm^3^	Amp B and itraconazole/No	NR
51	Anugulruengkitt et al. (2023) [2021]	Nakhon Si Thammarat [S]	*L*. *orientalis* [CL]	LM, PCR, DNA sequencing	Female; 1.5 years-old; NA; Immunocompetent	Itraconazole/No	NR

*Abbreviations*: Amp B, amphotericin B; BM, bone marrow; CL, cutaneous leishmaniasis; DAT, direct agglutination test; ID, identification number; LM, light microscopy; MCL, mucocutaneous leishmaniasis; NA, not available; NR, not reported; PCR, polymerase chain reaction; VL, visceral leishmaniasis; [C], Central region; [E], Eastern region; [N], Northern region; [S], Southern region; [W], Western region**.**

a In Mano et al. (2023a, b) and Jariyapan et al. (2025).

**Table 4:** Prevalence, incidence, epidemiological characteristics, and factors associated with the occurrence of leishmaniasis in Thailand (2004–2025).

ID	Reference [Study year]	Province [Region]	Population examined/Confirmed cases (*n*) or incidence data	Species distribution (% Prevalence or incidence)	Diagnostic methods	Characteristics of the study population (sex, age, occupation/immune status)	Reported/suggested factors associated with disease occurrence
**Cross-sectional study**							
52	Manomat e al. (2017) [2015–2016]	Trang [S]	724 HIV-positive individuals; *Leishmania* infection 25.1%	*L*. *orientalis* (identified as *L*. *siamensis*) (40.8%), *L*. *martiniquensis* (26.5%), *L*. *donovani* complex (20.4%), *L*. *lainsoni* (10.2%), *L*. *major* (2.1%)	DAT, nested ITS1 PCR, DNA sequencing	HIV-positive adults (> 18 years) (mean age 43.6 ± 8.5 years); Male and female (∼1:1); on ART follow-up; varied occupations (agriculture common); majority CD4^+^ > 500 cells/μl	Living in stilt houses (independent risk factor); CD4^+^ < 500 cells/μl (especially < 200 cells/μl); detectable HIV viral load (associated with PCR positivity); non-injection drug use (associated with seropositivity); possible underdiagnosis because asymptomatic infection was common
56	Jundang et al. (2024) [2020–2021]	Satun [S]	650 HIV-positive individuals; *Leishmania* infection 8.6%	*L*. *orientalis* (70.0%), *L*. *martiniquensis* (20.0%), *L*. *donovani* complex (10.0%)	DAT, nested ITS1 PCR, DNA sequencing	HIV-infected adults ≥ 18 years (mean age 43.6 ± 10.6 years); Male and female (∼1:1); Most on HAART (∼99%); Varied occupations (labor common); Majority CD4 ≥ 500 cells/mm^3^; Most viral load < 50 copies/ml	Increasing age; intravenous drug use; CD4 count < 500 cells/mm^3^; viral load ≥ 50 copies/ml; other factors (sex, education, occupation, opportunistic infection, bednet use, animal raising, housing type) not independently associated after multivariate adjustment
57	Piyaraj et al. (2024) [Jun-Aug 2022 (baseline), Nov-Dec 2022 and July-Aug 2023 (follow-up)]	Trang [S]	500 blood donors; *Leishmania* infection 19.0%	*L*. *martiniquensis* (98.5%), *L*. *donovani* complex (1.5%)	DAT, nested ITS1 PCR, DNA sequencing	Immunocompetent blood donors ≥ 18 years (mean age 37.1 ± 10.9 years); Male and female (∼1:1); Varied occupations (government officers, farmers, merchants, employees, and students); HIV-, HBV-, HCV-, and syphilis-negative per national blood bank screening; Without significant underlying immunosuppression	Living in stilt houses independently associated with infection; dog ownership significant in univariate analysis but not independently associated after adjustment; other factors (sex, age, occupation, drug use, travel history, underlying diseases, bednet use, proximity to forest, plantation, corral, nighttime outdoor activities) not significantly associated; environmental exposure under stilt houses suggested as favorable for sand fly vectors; high prevalence of asymptomatic carriage suggests potential transfusion transmission risk
54	Sriwongpan et al. (2021) [Sep-Nov 2015]	Chiang Rai [N]	392 immunocompetent individuals; *Leishmania* infection 7.1%	*L*. *martiniquensis* (4.8%), *L*. *orientalis* (57.1%), *Leishmania* spp. (38.1%)	DAT, nested ITS1-PCR, DNA sequencing	Immunocompetent adults; Male and female (∼2:1); Mean age 30.78 years in infected group and 19.40 years in non-infected group; Majority of students (79.8%), predominantly hill tribe populations, most without comorbidities, residents from both urban vocational school settings and rural mountainous communities	Female sex; increasing age; animal enclosures within 200 m of house; domestic animals in housing area; exposure to termite mounds; dark housing (urban site); not avoiding exposure to carrier animals; rural residence (mountainous area); evidence of infected sand flies (*P*. *stantoni*, *S. gemmea*) and black rats (possible reservoir)
**Case-control study**							
53	Charoensakulchai et al. (2020) [2015–2016]	Trang [S]	526 HIV-positive individuals; *Leishmania* infection 12.0%	*L*. *orientalis* (identified as *L*. *siamensis*) (12.0%), *L*. *martiniquensis* (4.6%), *L*. *donovani complex* (4.4%)	DAT, nested ITS1 PCR, DNA sequencing	HIV-infected adults ≥ 18 years attending HIV clinic (mean age 43.85 years); Male and female (∼1:1); Varied occupations	Intravenous drug use (associated with *L. orientalis*, possible transmission *via* shared syringes); female sex (*L. martiniquensis*; possibly related to differential vector exposure); recreational drug use; presence of comorbidities (immune dysregulation); opportunistic infections; CD4^+^ 200–500 cells/mm^3^; not using insect repellent (increased vector exposure); underlying immunosuppression in HIV-infected individuals; vector exposure in endemic areas
**Cohort study**							
55	Bualert et al. (2024) [2015–2016 (baseline), 2018–2019 (follow-up)]	Trang [S]	643 HIV-positive individuals at baseline, 506 followed up; Cumulative incidence 3.2%; Cumulative persistence 3.7%	Incidental species: *L. orientalis*; Persistent species: *L. donovani* complex; Baseline-positive species included *L. orientalis*, *L. martiniqu*ensis, *L. donovani* complex, *L. lainsoni*, *Leishmania* spp.	DAT, nested ITS1 PCR, DNA sequencing	HIV-infected adults ≥ 18 years (mean ∼44 years); Male and female (∼1:1); All asymptomatic and on HAART; Varied occupations (agriculture common); Majority undetectable viral load; Varied CD4 levels	Increasing age associated with incident infection; no significant association with sex, CD4 count, viral load, housing, animal exposure, or bednet use; asymptomatic infection was common; ongoing endemic transmission suggested

*Abbreviations*: DAT, direct agglutination test; ID, identification number; NR, not reported; PCR, polymerase chain reaction; [N], Northern region; [S], Southern region.

#### 3.3.2 Prevalence and incidence patterns

Autochthonous leishmaniasis cases were continuously reported in Thailand throughout the study period. Early reports described sporadic cases of VL, initially identified in immunocompetent individuals. However, from 2010 onwards, an increasing proportion of cases occurred among PLHIV, particularly those with low CD4^+^ T-cell counts (< 200 cells/mm^3^) (Suankratay et al., 2010; Bualert et al., 2012; Chusri et al., 2012; Phumee et al., 2013; Chiewchanvit et al., 2015; Supsrisunjai et al., 2017; Srivarasat et al., 2022). Across all studies, *L. martiniquensis* and *L. orientalis* were the most frequently detected species, followed by members of the *L. donovani* complex (*L. infantum*), while other species such as *L. lainsoni* and *L. major* were reported less frequently (Manomat et al., 2017). Relapse was documented in several cases, particularly among PLHIV, and amphotericin B was the drug of choice for treatment (Sukmee et al., 2008; Chusri et al., 2012; Osatakul et al., 2014; Pothirat et al., 2014; Chiewchanvit et al., 2015). Population-based studies further demonstrated that leishmaniasis in Thailand is not limited to sporadic clinical cases but shows characteristics of endemic transmission in specific regions. Among PLHIV, prevalence ranged from 8.61% to 25.1% in southern provinces, specifically in Trang and Satun, highlighting a geographically localized finding (Manomat et al., 2017; Charoensakulchai et al., 2020; Jundang et al., 2024), with cumulative incidence reported at 3.2% in a longitudinal study (Bualert et al., 2024). In contrast, studies in immunocompetent populations revealed prevalence rates of 7.1% in northern Thailand (Sriwongpan et al., 2021) and as high as 19.0% among blood donors in southern Thailand (Piyaraj et al., 2024). Asymptomatic infections were frequently detected across multiple studies involving both PLHIV and immunocompetent participants.

#### 3.3.3 Geographical occurrence and epidemiological patterns

**Fig. 3:**
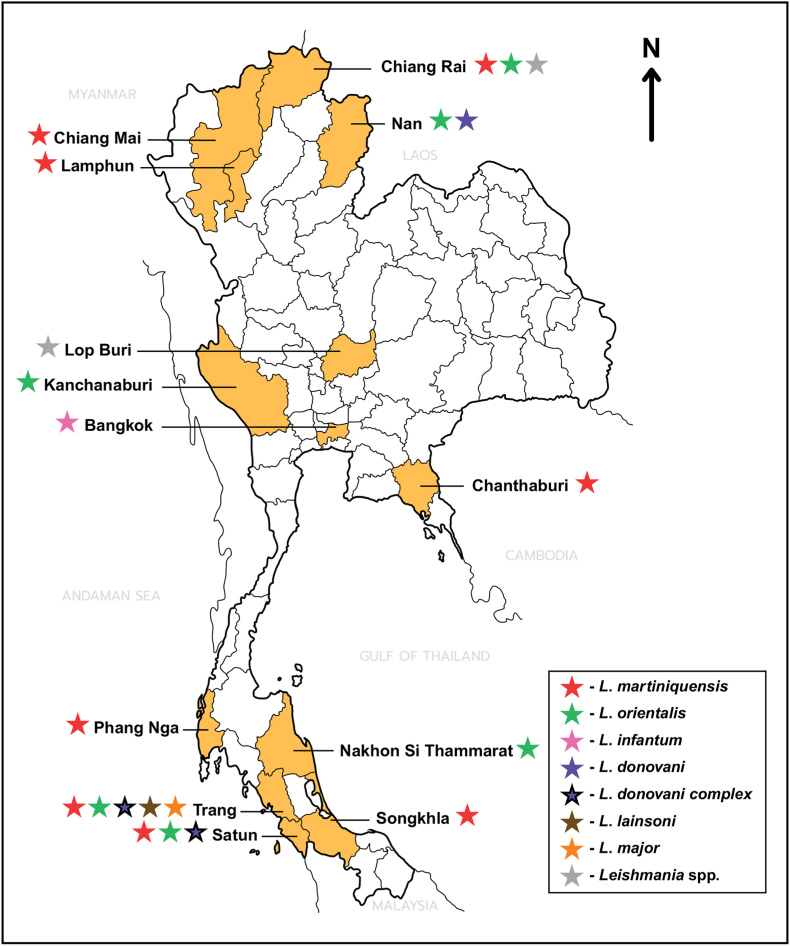
Occurrence of *Leishmania* species reported in Thailand from 2004 to 2025 according to data presented in Table 3, Table 4 The map summarizes province-level findings from included leishmaniasis studies.

Reported leishmaniasis cases and epidemiological studies were primarily concentrated in southern and northern Thailand. In southern Thailand, particularly Trang Province, both clinical case reports and high prevalence studies in PLHIV and blood donors were reported (Piyaraj et al., 2024). Autochthonous cases and evidence of ongoing transmission were also reported in other southern provinces, including Songkhla, Nakhon Si Thammarat, and Satun (Chusri et al., 2012; Osatakul et al., 2014; Anugulruengkitt et al., 2023; Jundang et al., 2024). In northern Thailand, provinces such as Nan, Chiang Rai, Lamphun, and Chiang Mai reported both symptomatic and asymptomatic infections (Kongkaew et al., 2007; Pothirat et al., 2014; Chiewchanvit et al., 2015; Jariyapan et al., 2018). Entomological studies in Chiang Rai discovered infected sandflies (*Phlebotomus stantoni*, *Sergentomyia gemmea*) and black rats as potential reservoirs (Sriwongpan et al., 2021). Central, eastern, and western regions reported sporadic autochthonous cases; however, large-scale prevalence studies remain limited in these areas (Maharom et al., 2008; Suankratay et al., 2010; Kattipathanapong et al., 2012; Supsrisunjai et al., 2017). The province-level occurrence of *Leishmania* species reported in Thailand from 2004 to 2025 is summarized in Fig. 3.

#### 3.3.4 Factors associated with leishmaniasis occurrence

Reported host-related factors included HIV infection, low CD4^+^ T-cell counts, HIV viral load, increasing age, and intravenous drug use. Immunosuppression was strongly associated with symptomatic disease and relapse (Charoensakulchai et al., 2020; Jundang et al., 2024). Environmental and behavioral factors were identified in multivariate analyses. Living in stilt houses was identified as an independent risk factor in multiple studies (Manomat et al., 2017; Piyaraj et al., 2024). Additional factors included proximity to animal enclosures, domestic animal ownership, exposure to termite mounds, dark housing conditions, rural or mountainous residence, and inadequate vector avoidance behaviors. Evidence of infected sand fly vectors and potential animal reservoirs supported zoonotic and vector-borne transmission pathways (Sriwongpan et al., 2021). Moreover, asymptomatic infection was a common finding across both PLHIV and immunocompetent populations (Manomat et al., 2017; Bualert et al., 2024).

### 3.4 Cross-disease synthesis of malaria and leishmaniasis in Thailand

**Fig. 4:**
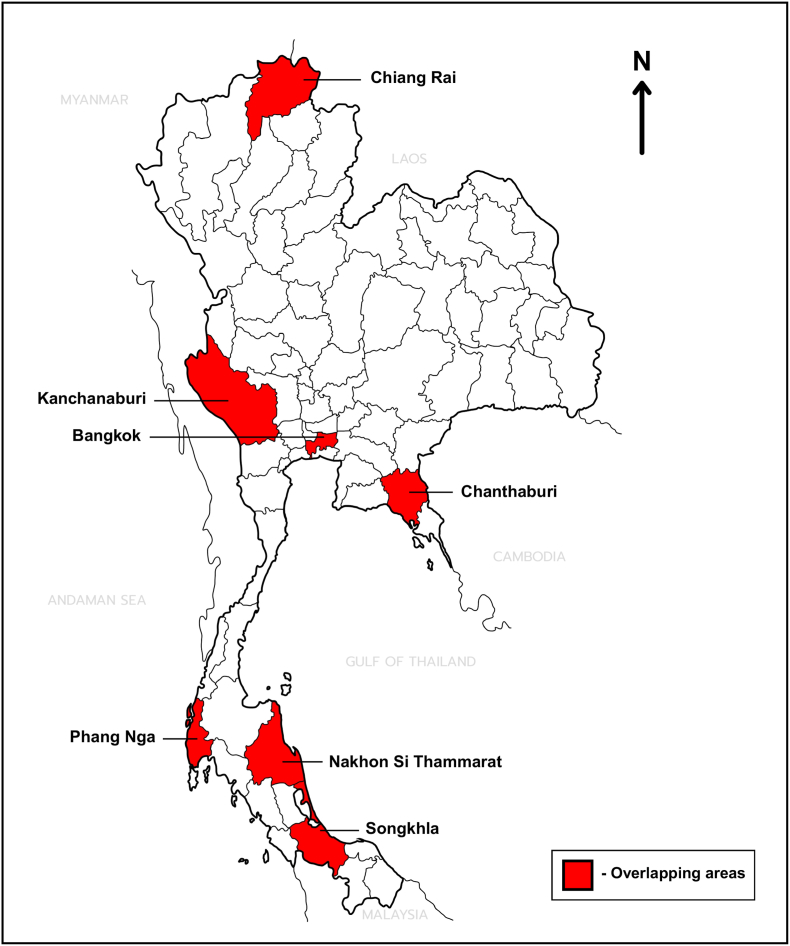
Provinces with reported occurrence of both malaria and autochthonous leishmaniasis in Thailand (2004–2025). Provinces highlighted in red indicate provinces where both diseases were independently reported in the included studies during the overall review period (Table 1, Table 2, Table 3, Table 4; Fig. 2, Fig. 3). This figure illustrates province-level co-occurrence based on aggregated reports from different study periods and does not imply confirmed co-infection, year-to-year temporal overlap, or shared transmission within the same populations.

According to data in Table 1, Table 2, Table 3, Table 4, seven provinces, including Phang Nga, Songkhla, Nakhon Si Thammarat (Southern region), Bangkok (Central region), Chanthaburi (Eastern region), Chiang Rai (Northern region), and Kanchanaburi (Western region), had reported occurrences of both malaria and leishmaniasis across the overall review period (Fig. 4). Although malaria surveillance data indicate a broader nationwide distribution, province-level evidence from included studies suggests co-occurrence at a relatively coarse spatial scale. Several of these provinces, particularly Chiang Rai, Kanchanaburi, and Chanthaburi, are border areas with documented malaria transmission and reported leishmaniasis cases. However, no confirmed malaria-*Leishmania* co-infection cases were identified in the reviewed studies. Therefore, the observed patterns reflect province-level co-occurrence rather than confirmed transmission within the same populations or ecological settings. Both diseases are associated with forested and plantation-based environments, which may provide shared ecological conditions for vectors and potential zoonotic reservoirs, supporting sustained transmission. Individuals engaged in forest-related activities, migrant workers, and marginalized populations may be at increased risk of exposure. Additionally, immunocompromised people (e.g. PLHIV) may be more susceptible to severe disease and potential undiagnosed. Forest-related occupations (e.g. rubber tappers, forest workers) and cross-border population mobility were consistently reported as important risk factors associated with increasing exposure in endemic areas (Table 5).

**Table 5:** Cross-disease synthesis of malaria and leishmaniasis in Thailand: provinces with reported occurrence of both diseases, ecological drivers, at-risk populations, and occupation and mobility-related exposures.

Feature	Cross-disease synthesis
Provinces with reported occurrence of both diseases	Seven provinces with reported occurrence of both diseases across the overall review period: Phang Nga, Songkhla, and Nakhon Si Thammarat (Southern region); Bangkok (Central region); Chanthaburi (Eastern region); Chiang Rai (Northern region); and Kanchanaburi (Western region)
Ecological drivers	Both diseases have been commonly reported in association with forested and plantation-based ecosystems, which provide shared ecological conditions for vectors and potential zoonotic reservoirs
At-risk populations	Populations potentially at increased risk include migrant workers, displaced and marginalized communities, individuals with frequent forest exposure, and immunocompromised individuals (e.g. PLHIV)
Occupation and mobility-related exposures	Forest-related occupations (e.g. rubber tappers, forest workers) and cross-border population mobility were consistently identified as potential exposure-related factors

## 4 Discussion

This systematic review synthesizes two decades of evidence on the prevalence, and epidemiological characteristics of malaria and leishmaniasis in Thailand. The findings demonstrate that despite Thailand’s substantial progress toward malaria elimination, persistent transmission foci, asymptomatic reservoirs, and the emergence of zoonotic *Plasmodium* species continue to challenge elimination goals. In parallel, leishmaniasis, previously regarded as a rare or imported disease, has shown sustained autochthonous transmission, expanded geographical range, and increased clinical diversity, supporting its reclassification as an endemic infection in Thailand. Although these two vector-borne diseases differ in historical endemicity and transmission dynamics, evidence from the included studies indicates province-level co-occurrence, together with shared ecological, occupational, and cross-border determinants.

It is important to note that the evidence base is not balanced across the two diseases. Malaria transmission patterns are informed by systematic, nationwide surveillance that captures trends across all 77 provinces. In contrast, the documented distribution of leishmaniasis is likely underestimated, as it relies on focal research efforts and clinical recognition of a relatively ‘new’ endemic threat. Consequently, the reported ‘absence’ of leishmaniasis in specific regions should be interpreted with caution, as it may reflect a lack of targeted screening rather than true non-endemicity. This asymmetry in the evidence base may influence the apparent province-level co-occurrence between the two diseases, potentially leading to overestimation in provinces with active leishmaniasis surveillance and reporting (e.g. Trang) and underestimation in areas where systematic screening is lacking.

Malaria transmission in Thailand from 2004 to 2025 remained heavily concentrated along international borders, particularly the Thailand-Myanmar, Thailand-Malaysia and Thailand-Cambodia borders. These patterns align with national surveillance data and regional assessments showing that border areas characterized by intense population mobility, forested landscapes, and limited healthcare access, remain malaria hotspots in the Greater Mekong Subregion (Geng et al., 2019; Karnchaisri et al., 2024; Pratumchart et al., 2025). *Plasmodium vivax* was the dominant species in most prevalence studies, particularly after 2012, accounting for more than half of malaria infections in multiple large-scale surveys. This transition reflects the success of artemisinin-based combination therapies and intensified malaria control programmes targeting *P. falciparum*. However, the biological characteristics of *P. vivax*, including hypnozoite-induced relapse, asymptomatic carriage, and lower detectability by routine diagnostics, pose a substantial obstacle to elimination. As documented in Thailand and other GMS settings, asymptomatic and recurrent *P. vivax* infections among migrants, forest workers, and border populations constitute a persistent silent reservoir capable of sustaining transmission despite low reported incidence (Howes et al., 2016; Villasis et al., 2022). Also, zoonotic malaria represents an additional challenge to malaria elimination in Thailand. Although zoonotic infections account for a relatively small proportion of reported cases, *P. knowlesi* has been sporadically detected across central, eastern, and southern Thailand, with clustering in provinces bordering Malaysia. Molecular surveys have further identified *P. cynomolgi*, *P. inui*, and mixed simian-human infections, indicating that the diversity of simian malaria parasites circulating in Thailand is broader than previously recognized (Putaporntip et al., 2021, 2022; Hmaidee et al., 2025).

The epidemiological patterns observed in Thailand are broadly consistent with those in neighboring countries (Myanmar, Lao PDR, Cambodia, and Malaysia), supporting the regional context of our findings. Across the Greater Mekong Subregion, declining transmission has been accompanied by a shift from *P. falciparum* to *P. vivax* predominance, as reported in Myanmar, Lao PDR, and Cambodia (Sandfort et al., 2020; Byrne et al., 2022; Legendre et al., 2023). Residual transmission in these settings is increasingly focal and concentrated in forested and border areas, where asymptomatic and submicroscopic infections act as persistent reservoirs, particularly among mobile and forest-exposed populations (Chaumeau et al., 2019; Byrne et al., 2022; Schneider et al., 2023; Rotejanaprasert et al., 2024). Myanmar remains an important source of cross-border transmission into Thailand, with sustained *P. falciparum* circulation in forested and conflict-affected areas (Legendre et al., 2023) whereas Cambodia shows similar challenges to Thailand, with *P. vivax* posing a major barrier to elimination due to relapse and asymptomatic carriage (Siv et al., 2016; Sandfort et al., 2020). Malaysia has reported zero indigenous *P. falciparum* and *P. vivax* malaria cases for seven consecutive years. However, malaria epidemiology in Malaysia is now dominated by zoonotic transmission, especially *P. knowlesi* (Raja et al., 2020; Lai et al., 2021). Recently, a systematic review by Ahebwa et al. (2025) has identified male gender, adult age, forest-related occupations, mobility, and widespread misdiagnosis as consistent determinants of zoonotic malaria, with macaque species, such as *Macaca nemestrina* and *M. fascicularis*, serving as major reservoirs for *P. knowlesi*, *P. inui*, and *P. cynomolgi*. Recent entomological evidence has suggested that *Anopheles* mosquitoes within the *Leucosphyrus* Group may serve as competent vectors for zoonotic simian malaria, potentially facilitating ongoing transmission of *P. inui* and *P. cynomolgi* between vectors and macaque hosts (Jeyaprakasam et al., 2023).

These infections were consistently associated with forest activities, agricultural or plantation work, and proximity to macaque habitats, demonstrating the importance of zoonotic spillover at the human-animal-environment interface. It should be noted that *P. knowlesi* is frequently misdiagnosed due to its morphological similarities to other species. It resembles the early trophozoite stages of *P. falciparum* and the later trophozoite and schizont stages of *P. malariae* (Lee et al., 2009). Additionally, previous studies have documented human infections with *P. cynomolgi* that were initially misidentified as *P. vivax* by microscopy but were later confirmed by PCR (Sai-ngam et al., 2022). The frequent misdiagnosis of *P. knowlesi* by light microscopy suggests that the true burden of zoonotic malaria may be underestimated (Singh and Daneshvar, 2013; Imwong et al., 2019; Putaporntip et al., 2021, 2022; Karnchaisri et al., 2024).

In contrast to malaria, leishmaniasis has historically been considered an imported or rare disease in Thailand. However, evidence synthesized from the present review indicates sustained autochthonous transmission across northern, southern, and central regions of the country. *Leishmania martiniquensis* and *L. orientalis* have emerged as the predominant causative agents. Prevalence studies further revealed substantial levels of asymptomatic or subclinical infection, especially among PLHIV in southern Thailand (Manomat et al., 2017; Jundang et al., 2024), suggesting that clinically recognized cases represent only a small fraction of the true disease burden. Across studies, infection risk has been consistently associated with environmental and housing-related factors, such as stilt houses, proximity to animal enclosures, termite mounds, and domestic animals, that facilitate sand fly breeding and sustained human-vector contact. Specifically, termite mounds have been identified as an independent risk factor for leishmaniasis since they serve as ideal breeding and resting sites for sand fly vectors. Examples are *P. argentipes* and *S. punjabensis* in Sri Lanka (Wijerathna and Gunathilaka, 2020) and *P. martini* in East Africa (Hassaballa et al., 2021). Termite mounds provide stable micro-environmental conditions, such as humidity, regulated temperature, and protection from environmental stressors, that support the survival and development of immature sand fly stages (Wijerathna and Gunathilaka, 2020; Hassaballa et al., 2021). These conditions have similarly been identified as key risk factors for ongoing transmission in other endemic regions (Calderon-Anyosa et al., 2018; O'Brien et al., 2024). These findings indicate that leishmaniasis in Thailand is now established as an endemic disease with complex eco-epidemiological profiles. It is consistent with global trends in which ecological change, host vulnerability, and parasite adaptation drive the emergence in non-classical endemic areas (Riebenbauer et al., 2024).

The coexistence of *L. martiniquensis* and *L. orientalis* underscores an underexplored issue in global leishmaniasis epidemiology, that is, the emergence of regionally adapted *Leishmania* species with overlapping but non-identical transmission cycles. Evidence suggests that *L. martiniquensis* may have a broader host range and zoonotic potential, supported by reports of infection in rodents, domestic animals, and multiple sand fly species (Kongkaew et al., 2007; Sriwongpan et al., 2021). In contrast, the transmission ecology of *L. orientalis* remains poorly defined. The identification of competent vectors responsible for the transmission of *L. martiniquensis* and *L. orientalis* remains unresolved. While phlebotomine sand flies are classically recognized as vectors of *Leishmania* spp. (Shaw, 2025), definitive evidence identifying competent vectors for *L. martiniquensis* and *L. orientalis* in Thailand is still lacking. Experimental studies demonstrated that *L. orientalis* could establish infection in *Culicoides sonorensis*, a permissive biting midge vector, whereas no infection was observed in *Lutzomyia longipalpis* sand flies (Chanmol et al., 2019). Complementing these findings, natural infection with *L*. *martiniquensis* has been detected in *C. peregrinus* (Diptera: Ceratopogonidae), suggesting that biting midges may serve as potential vectors for *Mundinia*-associated leishmaniasis (Kaewmee et al., 2023). However, vector competence and transmission capability under natural conditions have not yet been conclusively demonstrated. Given that *Mundinia* species exhibit ecological characteristics distinct from classical *Leishmania*, comprehensive entomological and reservoir investigations are essential. These uncertainties highlight the importance of a One Health approach, as the transmission of *Leishmania* in Thailand likely involves complex interactions among human, animal, and vector populations. The presence of multi-species endemicity in Thailand therefore calls for flexible, ecology-informed control strategies tailored to emerging *Leishmania* species rather than reliance on paradigms derived from traditional endemic settings. Despite these complexities, advances in molecular diagnostics with high sensitivity and specificity are now available and provide critical tools for surveillance and species differentiation in Thailand (Jariyapan et al., 2021; Mano et al., 2025).

One of the most important findings of this review is identification of seven provinces with reported occurrences of both malaria and leishmaniasis across the overall review period. These provinces several ecological and socio-economic characteristics, including forested landscapes, plantation-based livelihoods, border proximity, population mobility, and marginalized communities. However, because the included studies were conducted during different study periods and varied in geographical coverage, these findings should be interpreted as province-level co-occurrence based on aggregated evidence rather than confirmed year-to-year temporal overlap, co-infection, or transmission within the same populations or local transmission settings. Although direct evidence of *Plasmodium*-*Leishmania* co-infection in Thailand remains limited, individuals residing or working in these areas may experience repeated exposure to both *Anopheles* mosquitoes and leishmaniasis vectors, especially during evening outdoor activities (Esayas et al., 2024; Msellemu et al., 2024). While malaria and leishmaniasis differ in transmission dynamics and parasite biology, accumulating evidence indicates that HIV infection increases susceptibility to both diseases through impaired host immunity. In leishmaniasis, effective parasite control depends on Th1-mediated cellular immunity (e.g. IFN-γ and IL-12) required for macrophage activation and intracellular parasite killing. This response is markedly compromised in PLHIV due to CD4^+^ T-cell depletion and cytokine dysregulation (Costa-da-Silva et al., 2022), and immunocompromising conditions may further increase susceptibility and exacerbate clinical outcomes in cases of concurrent or sequential infections (Silva-Freitas et al., 2025). A similar immunological impairment has been described in malaria, where HIV-associated CD4^+^ T-cell depletion and immune dysregulation reduce parasite clearance and impair the acquisition and maintenance of effective antimalarial immunity (Figueroa-Romero et al., 2024). Consistent with these findings, studies from endemic areas along the Thailand-Myanmar border have shown that PLHIV have higher parasite densities and an increased risk of severe malaria compared to HIV-negative individuals (Rattanapunya et al., 2015). These findings suggest that HIV may represent a shared risk factor for both leishmaniasis and malaria by impairing cell-mediated and regulatory immune responses, consequently increasing susceptibility and disease severity in settings where both diseases have been reported. This pattern also underscores potential diagnostic and surveillance gaps, as febrile illnesses in malaria-endemic settings may be presumptively attributed to malaria, leading to delayed recognition of leishmaniasis, particularly cutaneous or atypical forms (Stoler and Awandare, 2016). As malaria elimination progresses and case numbers decline, other vector-borne diseases occupying similar ecological niches may become increasingly visible. In this post-elimination context, however, diagnostic overshadowing may persist, whereby healthcare providers continue to prioritize malaria in the differential diagnosis of febrile illness, potentially delaying recognition of other infections such as leishmaniasis.

Human *Plasmodium*-*Leishmania* co-infection has been documented for several decades. The earliest report originated from Sudan between 1933 and 1936, when up to 30% of patients with VL were infected with malaria (Henderson, 1937). Since then, natural co-infections have been reported across Africa and Asia, including Sudan, Ethiopia, Kenya, Uganda, India, Nepal, and Malaysia (reviewed by Ornellas-Garcia et al., 2023). In Thailand, the presence of macaque reservoirs associated with simian malaria and animal reservoirs implicated in *Leishmania* transmission further illustrates the ecological complexity of shared transmission environments involving both diseases (Bertram et al., 2024; Tiffin et al., 2025).

Although asymptomatic infection is highlighted as a shared challenge, its definition and epidemiological implications differ between malaria and leishmaniasis. In malaria, asymptomatic infection typically refers to the presence of circulating parasites (parasitemia) without overt clinical symptoms, particularly in individuals without recent antimalarial treatment. These infections are highly prevalent in malaria-endemic areas and are frequently detected in individuals who have developed partial immunity because of repeated exposure. Asymptomatic carriers are well-recognized as a significant silent reservoir contributing to sustaining transmission because asymptomatic carriers do not seek treatment, allowing the parasites to persist in their blood and be transmitted to mosquitoes (Mazigo et al., 2025). In contrast, asymptomatic leishmaniasis generally describes individuals with evidence of infection in the absence of clinical disease, often identified through serological or molecular methods. However, the role of such individuals in sustaining transmission remains less clearly defined and may vary depending on parasite species, host immunity, and vector dynamics (Pederiva et al., 2023). Therefore, while both conditions involve subclinical infection, their implications for disease control and surveillance are not directly comparable. This distinction further highlights the limitations of disease-specific surveillance approaches. For malaria, residual transmission driven by asymptomatic carriers of *P. vivax* and zoonotic parasites necessitates enhanced molecular surveillance, radical cure strategies, and strengthened cross-border collaboration. Reliance on microscopy alone is insufficient to detect low-density and simian infections (Putaporntip et al., 2021, 2022; Cui et al., 2022; Karnchaisri et al., 2024). For leishmaniasis, control relies primarily on early detection, vector surveillance, and reservoir management.

Ultimately, this review highlights the interconnected roles of human, animal, and environmental factors in the transmission of both diseases. Specifically, zoonotic reservoirs (e.g. macaques for simian malaria and black rats as potential animal reservoirs for *Leishmania*), vector ecology, and shared environmental conditions such as forested and plantation areas were consistently identified across studies, alongside human behavioral risk factors. These shared determinants underscore the importance of integrated, cross-disease surveillance approaches within a One Health framework, particularly in areas where both diseases have been reported. Future studies incorporating finer-scale epidemiological data and sub-provincial analyses are needed to better characterize shared transmission patterns and potential individual-level co-exposure.

*Limitations*. This review integrates diverse study designs over a 20-year period, providing a comprehensive overview of malaria and leishmaniasis epidemiology in Thailand. However, in case-control studies, participants were selected based on known infection status. Such studies were valuable for identifying associated risk factors, parasite species distribution, and epidemiological patterns among confirmed cases, but they do not accurately reflect true population-level prevalence. Since cases were pre-identified, the proportion of infected individuals could not be interpreted as representative of the general community. Consequently, estimates derived from these studies may overestimate or misrepresent the actual burden of malaria in the broader population. Nevertheless, these studies provided important insights into transmission areas and key environmental and behavioral risk factors associated with infection.

A primary limitation of this review is the structural asymmetry in the evidence base. The malaria data are predominantly surveillance-oriented, providing a high degree of confidence in reported incidence declines. Conversely, leishmaniasis data are more limited and heterogeneous, often restricted to specialized medical centers or specific provinces like Trang and Chiang Rai. This heterogeneity limits our ability to compare the true population-level burden of the two diseases directly. The temporal overlap between malaria and leishmaniasis could not be assessed at a year-to-year level because the included studies covered different study periods. Therefore, the observed co-occurrence should be interpreted as aggregated evidence rather than confirmed concurrent transmission. Furthermore, variability in diagnostic methods across studies might have affected the accuracy and comparability of reported prevalence. Some studies relied primarily on light microscopy RDTs, or LAMP, which have lower sensitivity for detecting low-parasitemia infections compared with molecular techniques such as PCR-based methods. For leishmaniasis, most prevalence studies were conducted among PLHIV, potentially limiting generalizability to the general population. Another important limitation is the potential under-detection of asymptomatic *Leishmania* infections. Publication bias toward unusual or severe cases may also have influenced the apparent reported occurrence of both diseases.

Importantly, given the asymmetry in the evidence base between malaria and leishmaniasis, the apparent absence of reported leishmaniasis cases in many provinces should not be interpreted as a true absence of the disease, but rather as a reflection of fragmented and uneven surveillance systems. This limitation may contribute to under-detection in areas without active screening, while potentially inflating the apparent concentration of cases in provinces with more intensive or targeted surveillance. Province-level analysis may overestimate apparent co-occurrence and cannot confirm whether both diseases occur within the same populations or ecological settings. Consequently, the currently available data may not fully capture the true epidemiological extent of leishmaniasis or the extent of province-level co-occurrence in reported malaria and leishmaniasis cases in Thailand.

## 5 Conclusion

In summary, this systematic review of evidence from 2004 to 2025 demonstrates that while Thailand is progressing toward malaria elimination, the epidemiological landscape is increasingly complicated because of persistent *P. vivax* transmission, asymptomatic reservoirs, and the emergence of zoonotic simian malaria parasites. Simultaneously, leishmaniasis has transitioned from a perceived rarity to an established endemic disease, characterized by reported occurrence across multiple regions in Thailand and evidence of substantial subclinical infections, particularly among immunocompromised populations. A key finding of this review is the documented occurrence of both diseases at the provincial level, associated with shared ecological conditions, forest-related occupational exposures, and cross-border population mobility. Although confirmed malaria-*Leishmania* co-infections have not yet been documented in Thailand, these findings suggest the need for increased clinical awareness and strengthened surveillance in areas where both diseases have been reported. The lack of reported co-infections may partly reflect diagnostic overshadowing, in which malaria is prioritized in the diagnostic workup of febrile illness, thus delaying the detection of leishmaniasis. Ultimately, the convergence of these shared epidemiological and ecological characteristics underscores the limitations of disease-specific control programmes. To sustain progress toward malaria elimination while addressing the growing burden of leishmaniasis, Thailand should adopt integrated surveillance and control strategies grounded in a One Health framework. This approach should link human clinical data with veterinary and entomological surveillance to manage zoonotic reservoirs and environmental risk factors effectively. In provinces reporting both malaria and leishmaniasis, clinicians should consider leishmaniasis in the differential diagnosis of patients presenting with persistent febrile illness, compatible clinical manifestations, or relevant environmental and occupational exposures. Furthermore, because both infections may present with relapse or persistent asymptomatic infection in certain patient populations, surveillance strategies should extend beyond passive hospital-based reporting to include targeted active and molecular surveillance where appropriate.

## Ethical approval

This study was exempted by the ethics committee of the Faculty of Medicine, Chulalongkorn University (COE No. 043/2025).

## CRediT authorship contribution statement

**Warachaya Apajamjarut:** Methodology, Investigation, Formal analysis, Data curation, Visualization, Writing - original draft, Writing - review & editing. **Nalin Siriwongsanon:** Methodology, Investigation, Formal analysis, Data curation, Visualization, Writing - original draft, Writing - review & editing. **Patanin Tonpon:** Methodology, Investigation, Formal analysis, Data curation, Visualization, Writing - original draft, Writing - review & editing. **Lapatrada Boonlertworakul:** Methodology, Investigation, Formal analysis, Data curation, Visualization, Writing - original draft, Writing - review & editing. **Ruamporn Joosiriwong:** Methodology, Investigation, Formal analysis, Data curation, Visualization, Writing - original draft, Writing - review & editing. **Baby Kyi Soe:** Formal analysis, Data curation, Writing - original draft, Writing - review & editing. **Saowalak Kaewmee:** Data curation, Writing - review & editing. **Jassada Saingamsook:** Data curation, Writing - review & editing. **Catherine Walton:** Writing - review & editing. **Sung Jae Lee:** Writing - review & editing. **Chunqing Lin:** Writing - review & editing. **George Dimopoulos:** Writing - review & editing. **Narissara Jariyapan:** Conceptualization, Methodology, Investigation, Formal analysis, Data curation, Visualization, Funding acquisition, Project administration, Resources, Writing - original draft, Writing - review & editing.

## Statement on the use of AI-assisted technologies

The authors used QuillBot (https://quillbot.com/) only to improve the readability and quality of the English language in this manuscript. This tool was not used to generate scientific content, analyze data, or influence the interpretation of results. The authors take full responsibility for the content of this article.

## Funding

This study was supported by Ratchadapiseksompotch Fund, Faculty of Medicine, Chulalongkorn University, grant number RA-MF-02/69.

## Declaration of Competing Interests

The authors declare that they have no known competing financial interests or personal relationships that could have appeared to influence the work reported in this paper.

## Data Availability

All data generated or analyzed during this study are included in this published article and its supplementary files.
