# Supplementary material for: Epidemiology of malaria and leishmaniasis in Thailand (2004–2025): A systematic review
Source: Curr Res Parasitol Vector Borne Dis. 2026 May 15;9:100386. doi: 10.1016/j.crpvbd.2026.100386 (PMC13217504; doi:10.1016/j.crpvbd.2026.100386)
Supplement: Multimedia component 1 [file mmc1.pdf]

Supplementary Table S1. The list of 57 studies (36 malaria and 21 leishmaniasis) included in this systematic review.

ID	Reference	Title	Study design/studies	Study location
	**Malaria**			
1	Lwin et al. (2008)	Clinically uncomplicated *Plasmodium falciparum* malaria with high schizontaemia: a case report	CR	Northern
2	Luvira et al. (2009)	Cerebral venous sinus thrombosis in severe malaria	CR	Western
3	Rijken et al. (2011)	Chloroquine resistant vivax malaria in a pregnant woman on the western border of Thailand	CR	Northern
4	Changpradub and Mungthin (2014)	Severe vivax malaria: a case report and a literature review	CR	Northeastern
5	Boonyarangka et al. (2022)	Co-infection with *Plasmodium vivax* and COVID-19 in Thailand	CR	Central
6	Jongwutiwes et al. (2004)	Naturally acquired *Plasmodium knowlesi* malaria in human, Thailand	CR	Central
7	Nakaviroj et al. (2015)	An autochthonous case of severe *Plasmodium knowlesi* malaria in Thailand	CR	Eastern
8	Ngernna et al. (2019)	Case series of human *Plasmodium knowlesi* infection on the southern border of Thailand	CR	Southern
9	Sai-ngam et al. (2022)	Case series of three malaria patients from Thailand infected with the simian parasite, *Plasmodium cynomolgi*	CR	Southern
10	Chaveepojnkamjorn and Pichainarong (2004)	Malaria infection among the migrant population along the Thai-Myanmar border area	CC	Northern, Western
11	Putaporntip et al. (2009)	Differential prevalence of *Plasmodium* infections and cryptic *Plasmodium knowlesi* malaria in humans in Thailand	CS	Northern, Eastern, Southern
12	Jongwutiwes et al. (2011)	*Plasmodium knowlesi* malaria in humans and macaques, Thailand	CS, CH	Northern, Eastern, Southern
13	Kritsiriwuthinan and Ngrenngarmlert (2011)	Asymptomatic malaria infections among foreign migrant workers in Thailand	CS	Central
14	Kitvatanachai and Rhongbutsri (2012)	Malaria in asymptomatic migrant workers and symptomatic patients in Thamaka District, Kanchanaburi Province, Thailand	CS	Western
15	Sermwittayawong et al. (2012)	Human *Plasmodium knowlesi* infection in Ranong Province, southwestern border of Thailand	CS	Southern
16	Baum et al. (2015)	Submicroscopic and asymptomatic *Plasmodium falciparum* and *Plasmodium vivax* infections are common in western Thailand - molecular and serological evidence	CS	Northern
17	Kaewpitoon et al. (2015)	Malaria risk areas in Thailand border	LE	Northeastern
18	Rattanapunya et al. (2015)	Prevalence of malaria and HIV coinfection and influence of HIV infection on malaria disease severity in population residing in malaria endemic area along the Thai-Myanmar border	CH	Northern
19	Sermwittayawong et al. (2015)	Characterization of malaria infection at two border areas of Thailand adjoining with Myanmar and Malaysia. Southeast Asian J Trop Med Public Health. 46, 551–557	CS	Southern
20	Sriwichai et al. (2017)	Imported *Plasmodium falciparum* and locally transmitted *Plasmodium vivax*: cross-border malaria transmission scenario in northwestern Thailand	CS	Northern

ID	Reference	Title	Study design/studies	Study location
21	Sattabongkot et al. (2018)	Prevalence of asymptomatic Plasmodium infections with sub-microscopic parasite densities in the northwestern border of Thailand: a potential threat to malaria elimination	CS	Northern
22	Lawpoolsri et al. (2019)	Epidemiological profiles of recurrent malaria episodes in an endemic area along the Thailand-Myanmar border: a prospective cohort study	CH	Northern
23	Kotepui et al. (2019)	Prevalence of malarial recurrence and hematological alteration following the initial drug regimen: a retrospective study in Western Thailand	CH	Northern
24	Mercado et al. (2019)	Spatiotemporal epidemiology, environmental correlates, and demography of malaria in Tak Province, Thailand (2012-2015)	LE	Northern
25	Nguitragool et al. (2019)	Highly heterogeneous residual malaria risk in western Thailand	CH	Western, Central
26	Saita et al. (2019)	Spatial heterogeneity and temporal trends in malaria on the Thai-Myanmar Border (2012-2017): A retrospective observational study	CH	Northern
27	Yorsaeng et al. (2019)	Indigenous *Plasmodium malariae* Infection in an Endemic Population at the Thai-Myanmar Border	CH	Western
28	Chaivisit et al. (2020)	Modelling malaria incidence in the upper part of southern Thailand	CS	Southern
29	Shimizu et al. (2020)	Malaria cross-sectional surveys identified asymptomatic infections of *Plasmodium falciparum*, *Plasmodium vivax* and *Plasmodium knowlesi* in Surat Thani, a southern province of Thailand	CS	Southern
30	Putaporntip et al. (2021)	*Plasmodium cynomolgi* co-infections among symptomatic malaria patients, Thailand	CS	Northern, Northeastern, Eastern, Southern
31	Putaporntip et al. (2022)	Cryptic *Plasmodium inui* and *Plasmodium fieldi* infections among symptomatic malaria patients in Thailand	CS	Northern, Northeastern, Eastern, Southern
32	Pongsoipetch et al. (2024)	Mapping malaria transmission foci in Northeast Thailand from 2011 to 2021: approaching elimination in a hypoendemic area	CH	Northeastern
33	Gilder et al. (2025)	Submicroscopic malaria in pregnancy and associated adverse pregnancy events: A case-cohort study of 4,352 women on the Thailand-Myanmar border	CH	Northern
34	Pratumchart et al. (2025)	Mapping malaria in Thailand: A Bayesian spatio-temporal analysis of national surveillance data	LE	Northern, Northeastern, Eastern, Southern
35	Aung et al. (2025a)	Slide positivity, trends, and risk factors of febrile *Plasmodium vivax* malaria along the Thailand-Myanmar border, 2018-2023	LE	Northern
36	Aung et al. (2025b)	Malaria care-seeking behaviours and infection prevalence among short-term Myanmar migrants in Thailand	CS	Northern
	**Leishmaniasis**			
37	Kongkaew et al. (2007)	Autochthonous visceral leishmaniasis: a report of a second case in Thailand	CR	Northern
38	Maharom et al. (2008)	Visceral leishmaniasis caused by *Leishmania infantum* in Thailand	CR	Central
39	Sukmee et al. (2008)	A suspected new species of *Leishmania*, the causative agent of visceral leishmaniasis in a Thai patient	CR	Southern

ID	Reference	Title	Study design/studies	Study location
40	Suankratay et al. (2010)	Autochthonous visceral leishmaniasis in a human immunodeficiency virus (HIV)-infected patient: the first in Thailand and review of the literature	CR	Eastern
41	Bualert et al. (2012)	Autochthonous disseminated dermal and visceral leishmaniasis in an AIDS patient, southern Thailand, caused by *Leishmania siamensis*	CR	Southern
42	Chusri et al. (2012)	Consecutive cutaneous and visceral leishmaniasis manifestations involving a novel *Leishmania* species in two HIV patients in Thailand	CR	Southern
43	Kattipathanapong et al. (2012)	The first reported case of autochthonous cutaneous leishmaniasis in Thailand	CR	Central
44	Phumee et al. (2013)	Detection of *Leishmania siamensis* DNA in saliva by polymerase chain reaction	CR	Northern
45	Osatakul et al. (2014)	Recurrences of visceral leishmaniasis caused by *Leishmania siamensis* after treatment with amphotericin B in a seronegative child	CR	Southern
46	Pothirat et al. (2014)	First isolation of *Leishmania* from northern Thailand: case report, identification as *Leishmania martiniquensis* and phylogenetic position within the *Leishmania enriettii* complex	CR	Northern
47	Chiewchanvit et al. (2015)	Chronic generalized fibrotic skin lesions from disseminated leishmaniasis caused by *Leishmania martiniquensis* in two patients from northern Thailand infected with HIV	CR	Northern
48	Supsrisunjai et al. (2017)	Disseminated autochthonous dermal leishmaniasis caused by *Leishmania siamensis* (PCM2 Trang) in a patient from central Thailand infected with human immunodeficiency virus	CR	Western
49	Jariyapan et al. (2018)	*Leishmania* (*Mundinia*) *orientalis* n. sp. (Trypanosomatidae), a parasite from Thailand responsible for localised cutaneous leishmaniasis	CR	Northern
50	Srivarasat et al. (2022)	Autochthonous disseminated cutaneous, mucocutaneous, and visceral leishmaniasis caused by *Leishmania martiniquensis* in a patient with HIV/AIDS from northern Thailand and literature review	CR	Northern
51	Anugulruengkitt et al. (2023)	Simple nodular cutaneous leishmaniasis caused by autochthonous *Leishmania* (*Mundinia*) *orientalis* in an 18-month-old girl: the first pediatric case in Thailand and literature review	CR	Southern
52	Manomat el al. (2017)	Prevalence and risk factors associated with *Leishmania* infection in Trang Province, southern Thailand	CS	Southern
53	Charoensakulchai et al. (2020)	Risk factors of *Leishmania* infection among HIV-infected patients in Trang Province, southern Thailand: a study on three prevalent species	CC	Southern
54	Sriwongpan et al. (2021)	Prevalence and associated risk factors of *Leishmania* infection among immunocompetent hosts: a community-based study in Chiang Rai, Thailand	CS	Northern
55	Bualert et al. (2024)	Incidence and persistence of asymptomatic *Leishmania* infection among HIV-infected patients in Trang Province, Southern Thailand: a cohort study	CH	Southern
56	Jundang et al. (2024)	*Leishmania* infection among HIV-infected patients in a southern province of Thailand: a cross-sectional study	CS	Southern
57	Piyaraj et al. (2024)	Asymptomatic *Leishmania* infection among blood donors in a southern province of Thailand	CS	Southern

Supplementary Table S2. Risk of bias assessment of the 24 included case reports on the distribution of malaria and leishmaniasis, using the Joanna Briggs Institute (JBI) Critical Appraisal Checklist for Case Reports (Moola et al., 2020). Each domain was assessed as "Y = Yes," "N = No," or "U = Unclear."

ID	Reference	D1	D2	D3	D4	D5	D6	D7	D8	Total score*
	**Malaria**									
1	Lwin et al. (2008)	Y	Y	Y	Y	Y	Y	Y	Y	100
2	Luvira et al. (2009)	Y	Y	Y	Y	Y	Y	Y	Y	100
3	Rijken et al. (2011)	Y	Y	Y	Y	Y	Y	Y	Y	100
4	Changpradub and Mungthin (2014)	Y	Y	Y	Y	Y	Y	Y	Y	100
5	Boonyarangka et al. (2022)	Y	Y	Y	Y	Y	Y	Y	Y	100
6	Jongwutiwes et al. (2004)	Y	Y	Y	Y	Y	Y	Y	Y	100
7	Nakaviroj et al. (2015)	Y	Y	Y	Y	Y	Y	Y	Y	100
8	Ngernna et al. (2019)	Y	Y	Y	Y	Y	Y	Y	Y	100
9	Sai-ngam et al. (2022)	Y	Y	Y	Y	Y	Y	Y	Y	100
	**Leishmaniasis**									
37	Kongkaew et al. (2007)	Y	Y	Y	Y	Y	Y	Y	Y	100
38	Sukmee et al. (2008)	Y	Y	Y	Y	Y	Y	Y	Y	100
39	Maharom et al. (2008)	Y	Y	Y	Y	Y	Y	Y	Y	100
40	Suankratay et al. (2010)	Y	Y	Y	Y	Y	Y	Y	Y	100
41	Bualert et al. (2012)	Y	Y	Y	Y	Y	Y	Y	Y	100
42	Chusri et al. (2012)	Y	Y	Y	Y	Y	Y	Y	Y	100
43	Kattipathanapong et al. (2012)	Y	Y	Y	Y	Y	Y	Y	Y	100
44	Phumee et al. (2013)	Y	Y	Y	Y	Y	Y	Y	Y	100
45	Osatakul et al. (2014)	Y	Y	Y	Y	Y	Y	Y	Y	100
46	Pothirat et al. (2014)	Y	Y	Y	Y	Y	Y	Y	Y	100
47	Chiewchanvit et al. (2015)	Y	Y	Y	Y	Y	Y	Y	Y	100
48	Supsrisunjai et al. (2017)	Y	Y	Y	Y	Y	Y	Y	Y	100
49	Jariyapan et al. (2018)	Y	Y	Y	Y	Y	Y	Y	Y	100
50	Srivarasat et al. (2022)	Y	Y	Y	Y	Y	Y	Y	Y	100
51	Anugulruengkitt et al. (2023)	Y	Y	Y	Y	Y	Y	Y	Y	100

Domains (D)

1. Were patient's demographic characteristics clearly described?
2. Was the patient's history clearly described and presented as a timeline?
3. Was the current clinical condition of the patient on presentation clearly described?
4. Were diagnostic tests or assessment methods and the results clearly described?
5. Was the intervention(s) or treatment procedure(s) clearly described?
6. Was the post-intervention clinical condition clearly described?
7. Were adverse events (harms) or unanticipated events identified and described?
8. Does the case report provide takeaway lessons, i.e., location/region? (Data used to map distribution)

***Scoring and interpretation:**
Calculating score: (Total number of “Yes” responses ÷ Total number of appraisal items) × 100

- ≥ 70%: Low risk of bias
- 50-69: Moderate risk of bias
- < 50%: High risk of bias

Supplementary Table S3. Risk of bias assessment of the 28 included studies for prevalence and distribution of malaria and leishmaniasis, using the Joanna Briggs Institute (JBI) Critical Appraisal Checklist for Prevalence Studies (Munn et al., 2015). Each domain was assessed as "Y = Yes," "N = No," or "U = Unclear."

ID	Reference	D1	D2	D3	D4	D5	D6	D7	D8	D9	Total score*
	**Malaria**										
10	Chaveepojnkamjorn et al. (2004)	Y	Y	Y	Y	Y	Y	Y	Y	Y	100
11	Putaporntip et al. (2009)	Y	Y	Y	Y	Y	Y	Y	Y	Y	100
12	Jongwutiwes et al. (2011)	Y	Y	Y	Y	Y	Y	Y	Y	Y	100
13	Kritsiriwuthinan and Ngrenngarmlert (2011)	Y	Y	Y	Y	Y	Y	Y	Y	Y	100
14	Kitvatanachai and Rhongbutsri (2012)	Y	Y	Y	Y	Y	Y	Y	Y	Y	100
15	Sermwittayawong et al. (2012)	Y	Y	Y	Y	Y	Y	Y	Y	Y	100
16	Baum et al. (2015)	Y	Y	Y	Y	Y	Y	Y	Y	Y	100
17	Kaewpitoon et al. (2015)	Y	Y	Y	Y	Y	Y	Y	Y	Y	100
18	Rattanapunya et al. (2015)	Y	Y	Y	Y	Y	Y	Y	Y	Y	100
19	Sermwittayawong et al. (2015)	U	U	Y	U	Y	Y	Y	Y	Y	67
20	Sriwichai et al. (2017)	Y	Y	Y	Y	Y	Y	Y	Y	Y	100
21	Sattabongkot et al. (2018)	Y	Y	Y	Y	Y	Y	Y	Y	Y	100
23	Kotepui et al. (2019)	Y	Y	Y	Y	Y	Y	Y	Y	Y	100
24	Mercado et al. (2019)	Y	Y	Y	Y	Y	Y	Y	Y	Y	100
26	Saita et al. (2019)	Y	Y	Y	Y	Y	Y	Y	Y	Y	100
28	Chaivisit et al. (2020)	Y	Y	Y	Y	Y	Y	Y	Y	Y	100
29	Shimizu et al. (2020)	Y	Y	Y	Y	Y	Y	Y	Y	Y	100
30	Putaporntip et al. (2021)	Y	Y	Y	Y	Y	Y	Y	Y	Y	100
31	Putaporntip et al. (2022)	Y	Y	Y	Y	Y	Y	Y	Y	Y	100
32	Pongsoipetch et al. (2024)	Y	Y	Y	Y	Y	Y	Y	Y	Y	100
34	Pratumchart et al. (2025)	Y	Y	Y	Y	Y	Y	Y	Y	Y	100
35	Aung et al. (2025a)	Y	Y	Y	Y	Y	Y	Y	Y	Y	100
36	Aung et al. (2025b)	Y	Y	Y	Y	Y	Y	Y	Y	Y	100
	**Leishmaniasis**										
52	Manomat el al. (2017)	Y	Y	Y	Y	Y	Y	Y	Y	Y	100
53	Charoensakulchai et al. (2020)	Y	Y	Y	Y	Y	Y	Y	Y	Y	100

54	Sriwongpan et al. (2021)	Y	Y	Y	Y	Y	Y	Y	Y	Y	100
56	Jundang et al. (2024)	Y	Y	Y	Y	Y	Y	Y	Y	Y	100
57	Piyaraj et al. (2024)	Y	Y	Y	Y	Y	Y	Y	Y	Y	100

Domains (D)
1. Was the sample frame appropriate to address the target population?
2. Were study participants sampled in an appropriate way?
3. Was the sample size adequate?
4. Were the study subjects and the setting described in detail?
5. Was the data analysis conducted with sufficient coverage of the identified sample?
6. Were valid methods used for the identification of the condition?
7. Was the condition measured in a standard, reliable way for all participants?
8. Was there appropriate statistical analysis?
9. Was the response rate adequate, and if not, was the low response rate managed appropriately?

***Scoring and interpretation:**
Calculating score: (Total number of "Yes" responses ÷ Total number of appraisal items) × 100
- ≥ 70%: Low risk of bias
- 50-69: Moderate risk of bias
- < 50%: High risk of bias

Supplementary Table S4. Risk of bias assessment of the five included cohort studies for incidence and distribution of malaria and leishmaniasis,, using the Joanna Briggs Institute (JBI) Critical Appraisal Checklist for Cohort Studies (Moola et al., 2020). Each domain was assessed as "Y = Yes," "N = No," or "U = Unclear."

ID	Reference	D1	D2	D3	D4	D5	D6	D7	D8	D9	D10	D11	Total score*
	**Malaria**												
22	Lawpoolsri et al. (2019)	Y	Y	Y	Y	Y	Y	Y	Y	Y	Y	Y	100
25	Nguitragool et al. (2019)	Y	Y	Y	Y	Y	Y	Y	Y	Y	Y	Y	100
27	Yorsaeng et al. (2019)	Y	Y	Y	Y	Y	Y	Y	Y	Y	Y	Y	100
33	Gilder et al. (2025)	Y	Y	Y	Y	Y	Y	Y	Y	Y	Y	Y	100
	**Leishmaniasis**												
55	Bualert et al. (2024)	Y	Y	Y	Y	Y	Y	Y	Y	Y	Y	Y	100

Domains (D)

1. Were the two groups similar and recruited from the same population?
2. Were the exposures measured similarly to assign people to both exposed and unexposed groups?
3. Was the exposure measured in a valid and reliable way?
4. Were confounding factors identified?
5. Were strategies to deal with confounding factors stated?
6. Were the groups/participants free of the outcome at the start of the study (or at the moment of exposure)?
7. Were the outcomes measured in a valid and reliable way?
8. Was the follow up time reported and sufficient to be long enough for outcomes to occur?
9. Was follow up complete, and if not, were the reasons to loss to follow up described and explored?
10. Were strategies to address incomplete follow up utilized?
11. Was appropriate statistical analysis used?

***Scoring and interpretation:**

Calculating score: (Total number of "Yes" responses ÷ Total number of appraisal items) × 100

- ≥ 70%: Low risk of bias
- 50-69: Moderate risk of bias
- < 50%: High risk of bias
